# A Mediator-cohesin axis controls heterochromatin domain formation

**DOI:** 10.1038/s41467-022-28377-7

**Published:** 2022-02-08

**Authors:** Judith H. I. Haarhuis, Robin H. van der Weide, Vincent A. Blomen, Koen D. Flach, Hans Teunissen, Laureen Willems, Thijn R. Brummelkamp, Benjamin D. Rowland, Elzo de Wit

**Affiliations:** 1 Division of Cell Biology, The Netherlands Cancer Institute, Plesmanlaan 121, 1066 CX Amsterdam, The Netherlands; 2 Division of Gene Regulation, Oncode Institute, The Netherlands Cancer Institute, Plesmanlaan 121, 1066 CX Amsterdam, The Netherlands; 3 Division of Biochemistry, Oncode Institute, The Netherlands Cancer Institute, Plesmanlaan 121, 1066 CX Amsterdam, The Netherlands; 4 Present Address: Hubrecht Institute-KNAW, Utrecht, The Netherlands

**Keywords:** Epigenomics, Chromosomes, Epigenetics

## Abstract

The genome consists of regions of transcriptionally active euchromatin and more silent heterochromatin. We reveal that the formation of heterochromatin domains requires cohesin turnover on DNA. Stabilization of cohesin on DNA through depletion of its release factor WAPL leads to a near-complete loss of heterochromatin domains. We observe the opposite phenotype in cells deficient for subunits of the Mediator-CDK module, with an almost binary partition of the genome into dense H3K9me3 domains, and regions devoid of H3K9me3 spanning the rest of the genome. We suggest that the Mediator-CDK module might contribute to gene expression by limiting the formation of dense heterochromatin domains. WAPL deficiency prevents the formation of heterochromatin domains, and allows for gene expression even in the absence of the Mediator-CDK subunit MED12. We propose that cohesin and Mediator affect heterochromatin in different ways to enable the correct distribution of epigenetic marks, and thus to ensure proper gene expression.

## Abstract (web summary)

The link between 3D genome architecture and gene expression is still far from resolved. Here the authors show that loss of the CDK catalytic subunit of the Mediator complex results in heterochromatic silencing, which can be rescued by stabilization of cohesin on chromatin.

## Introduction

Within the nucleus active and inactive genomic regions segregate into euchromatin and heterochromatin. The latter is molecularly defined by the post-translational methylation of the ninth lysine of histone H3 (H3K9me)^1,2^. This histone modification creates a binding site for heterochromatin protein 1 (HP1). Recent results suggest that heterochromatin forms a phase-separated compartment inside the nucleus^3^. Heterochromatin has the propensity to self-amplify through the interaction of HP1 with the histone methyltransferase Suv39h, which methylates H3K9 and creates binding site for HP1^4^. How this amplification is kept in check remains unknown.

The distribution of heterochromatic histone marks shows a remarkable correlation with many genomic features. Examples include late replication^5^, lamina association^6^, and inactive compartments. Chromosomes are organized into multi-megabase domains called A and B compartments into which active and inactive chromatin is physically separated^7,8^. Cohesin-dependent chromatin loops counteract compartmentalization^9,10^, and loss of cohesin is associated with an increase in compartmentalization^9,11^.

The 3D genome has been heralded as an important factor in the regulation of gene expression. However, the total ablation of cohesin-dependent loops shows only mild overall effects on gene expression in cancer cell lines^12,13^. On the other hand, the loss of individual CTCF sites, through mutation or DNA methylation, can have severe pathological consequences in limb development^14^ and cancer progression^15^. Changes in the 3D genome may therefore have unexpected and cell-type-specific phenotypic effects.

In this work, we explore the role of chromatin loop formation and compartmentalization in the control of the epigenome and the expression of genes. We reveal a surprising link between the three-dimensional organization of the genome and the linear distribution of chromatin modifications. Stabilization of cohesin on chromatin, besides reducing compartmentalization, also counteracts heterochromatin domain formation. Loss of the Mediator complex CDK-module subunits MED12 and CCNC on the other hand increases both compartmentalization and heterochromatin domain formation. We conclude that a Mediator-cohesin axis controls both the spatial and the linear epigenome.

## Results and discussion

**Fig. 1:**
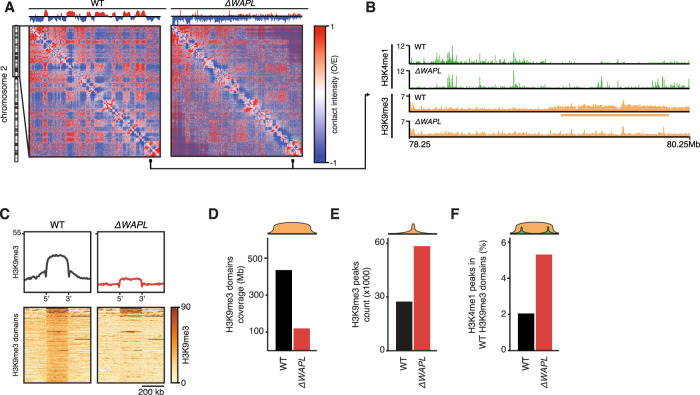
Stabilization of cohesin results in the loss of H3K9Me3 domains. **A** Observed over expected (O/E) Hi-C matrixplots at 100 kb resolution show the contact frequencies for wild-type (WT) and *∆WAPL* cells for the p-arm of chromosome 2. Contact matrices are visualized using GENOVA. Segregation of the genome into A and B compartments is quantified using the compartment score^11^, shown above the matrix. **B** ChIPseq tracks for H3K4me1 and H3K9me3 in WT and *∆WAPL* cells. Orange rectangle indicates the position of a H3K9me3 domain. **C** Alignment of H3K9me3 signal on H3K9me3 domains identified in WT H3K9me3 ChIPseq data. Average signal is shown on top, bottom shows heatmap of the raw signal. **D** Quantification of the genomic coverage of the H3K9me3 domains in WT and *∆WAPL* cells. **E** Quantification of the total number of H3K9me3 peaks in WT and *∆WAPL* cells. **F** Percentage of H3K4me1 peaks found in genomic regions that are identified as H3K9me3 peaks in WT cells.

We have previously shown that the stabilization of cohesin on chromatin has a major effect on the 3D genome. Loss of the cohesin-release factor WAPL leads to a genome-wide increase in the length and number of CTCF-anchored loops^9,16^. This in turn leads to a severe decrease in compartmentalization (Fig. 1A). We wondered whether the change in the 3D genome was associated with a change in the epigenome. To this end, we mapped core histone modifications H3K4me1 and H3K9me3 in wild-type and *WAPL* knock-out (*∆WAPL*) HAP1 cells. In wild-type cells, H3K9me3 shows broad domain-like distributions that overlap almost exclusively with B compartments (Supplementary Fig. 1a). In A compartments, H3K9me3 shows a more focal distribution, covering transposable elements (Supplementary Fig. 1b). In ∆*WAPL* cells, we see a near-complete loss of H3K9me3 domains (Fig. 1B–D) and a doubling of H3K9me3 peaks (Fig. 1E). We also see a marked increase of H3K4me1 peaks in genomic regions that are H3K9me3 domains in wild-type cells (3414 vs. 8310, Fig. 1F). WAPL-mediated cohesin-release apparently maintains a balance between eu- and heterochromatin domains.

**Fig. 2:**
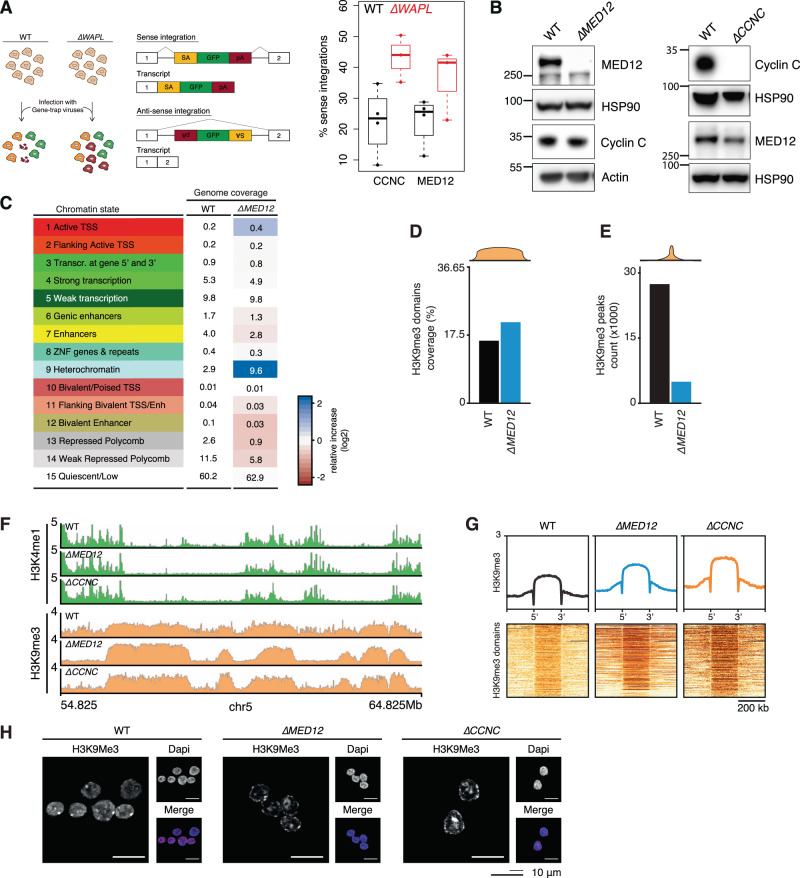
Mediator-CDK subunits restrict heterochromatin. **A** Left panel shows schematic explanation of the haploid genetic gene trap assay. Sense integrations with respect to a protein coding gene result in truncating deletion. Anti-sense integrations are largely inconsequential and serve as a control for accessibility of the gene. Right panel shows percentage of sense integrations (over a total of sense and anti-sense) shown for WT and *∆WAPL* cells for the Mediator subunits Cyclin-C (CCNC) and MED12. Boxplot shows the result of *n* = 3 haploid genetic gene trap assays for WT and *∆WAPL* cells. Boxes indicate the interquartile range (IQR) of the data (25–75%) and box center line indicates the median. Whiskers extend to the minimum or maximum value that lies no further than 1.5 times the IQR from the bottom or top of the box, respectively. **B** Western blot analysis confirming knock-out of MED12 and Cyclin-C. HSP90 and actin represent loading controls for MED12 and Cyclin-C panels directly above. Representative images are shown from experiments that have been performed twice. **C** ChromHMM analysis segmenting the genome in 15 chromatin states shown for WT and MED12 cells. Table shows the percentage of genome coverage. Red/blue color scale indicates the relative increase or decrease in *∆MED12* compared to the WT cells. **D** Barplot showing the coverage of H3K9me3 domains over the genome and (**E**) the absolute number of H3K9me3 peaks. **F** Example region showing the H3K4me1 (green) and H3K9me3 (orange) ChIPseq profiles in WT, *∆MED12* and *∆CCNC* cells. **G** Aggregate plots showing the average H3K9me3 signal over all the H3K9me3 domains for WT and mutant cells. Heatmaps show the signal in and around individual domains. **H** Immunofluorescence analysis of H3K9me3 levels in WT, *∆MED12* and *∆CCNC* cells. Representative images are shown from experiments that have been performed twice (see Supplementary Fig. 2e).

As *∆WAPL* cells almost completely lack heterochromatin domains, we reasoned that this could be an interesting setting to identify factors that restrict the amplification of heterochromatin domains. While wild-type cells presumably are dependent on such suppressors of heterochromatin, these factors may no longer be needed in *∆WAPL* cells. If so, loss of suppressors of heterochromatin should be detrimental to wild-type cells, but not to *∆WAPL* cells. To this end we revisited a haploid genetic screen we performed in wild-type and *∆WAPL* cells^9^. The screen is based on a viral gene trap assay that knocks out a gene when it lands in the sense orientation, whereas anti-sense integration leaves the gene unaffected (Fig. 2A). A decrease in the ratio of sense over anti-sense integrations is an indication that the gene is required for cell viability^17^. We found that disruption of genes encoding two subunits of the CDK-module of the Mediator complex, *MED12* and *Cyclin C* (*CCNC*), affected the proliferation of wild-type cells, but this growth defect was mitigated in *∆WAPL* cells (Fig. 2A). To study the role of *MED12* and *Cyclin C*, we generated knock-out cells using CRISPR-Cas9 genome editing. Western blot analysis (Fig. 2B), Sanger sequencing, and Targeted Locus Amplification (TLA)^18^ confirmed the perturbation of the genes (Supplementary Fig. 2A, B).

As MED12 and Cyclin C both are part of the same module of the Mediator complex^19^, loss of either of these factors presumably affects the same cellular pathway. To characterize the effect of mutating this module, we charted the epigenomic landscape by performing ChIPseq of 5 histone modifications^20^: H3K4me3, H3K4me1, H3K36me3, H3K27me3 and H3K9me3 in wild-type and *∆MED12* (Supplementary Fig. 2c). We performed ChromHMM analysis^21^ to segment the genome into 15 different chromatin states. The most obvious difference is that loss of MED12 leads to a more than three-fold increase in the heterochromatin state (Fig. 2C). When we inspected the genome-wide coverage of H3K9me3 domains in *∆MED12* cells, we saw only a mild increase (Fig. 2D). Surprisingly, we even saw a 5.5-fold decrease in the amount of H3K9me3 peaks (27403 vs. 4976, Fig. 2E, Supplementary Fig. 1C). Upon further inspection of the ChIPseq track we found that for both for *∆MED12* and *∆CCNC* there was a marked increase in the H3K9me3 signal at wild-type H3K9me3 domains (Fig. 2F), leading to more pronounced heterochromatin domains. The H3K4me1 signal, on the other hand, was diminished in heterochromatin domains in *∆MED12* and *∆CCNC* cells (Fig. 2F, Supplementary Fig. 2d). Whole-genome analysis of the H3K9me3 domain signals corroborated these results (Fig. 2G). To further assess the role of MED12 and Cyclin C in heterochromatin distribution, we performed immunofluorescence (IF) analysis of H3K9me3 and HP1α. Whereas in wild-type cells a low level of H3K9me3 can be found throughout the entire nucleus, in *∆MED12* and *∆CNCC* cells H3K9me3 levels are reduced to baseline levels in much of the nucleus, and seems to be accumulated at specific sites and the nuclear periphery (Fig. 2H). Quantification of the IF data shows a decrease in the median H3K9me3 signal strength confirming the redistribution of H3K9me3 in the nucleus (Supplementary Fig. 2E). The IF quantification for HP1α showed a similar result (Supplementary Fig. 2f). To assess whether canonical heterochromatin proteins were deregulated we performed RNAseq analysis. We did not observe differential expression for genes encoding classical heterochromatin proteins such as HP1-isoforms (Supplementary Fig. 3A), nor could we pinpoint any other gene expression differences that provide an indirect explanation for changes in heterochromatinization.

**Fig. 3:**
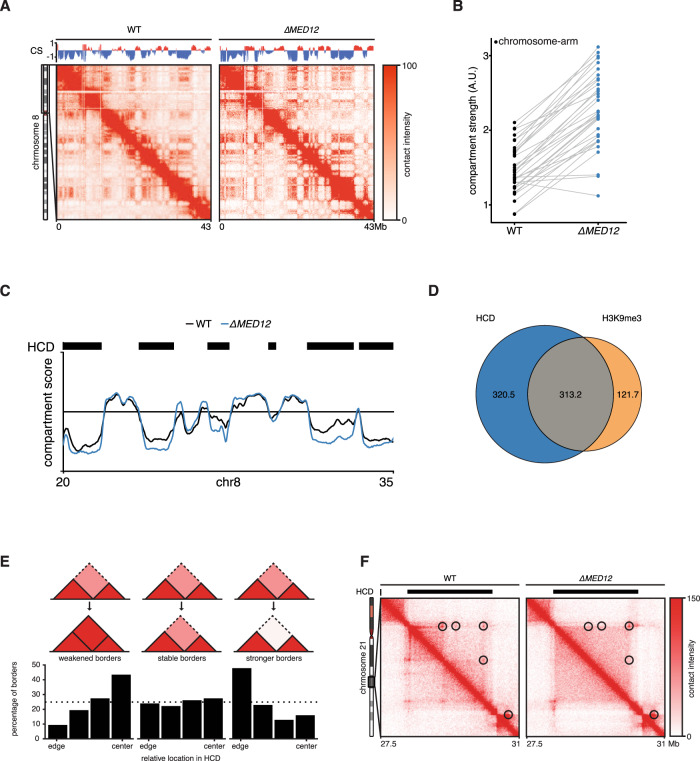
Loss of MED12 affects 3D genome organization. **A** ICE normalized Hi-C contact matrices at 100 kb resolution for WT and *∆MED12* are shown for the p arm of chromosome 8. Above the matrices the compartment score is plotted. Contact matrices are visualized using GENOVA. **B** The compartment strength is calculated for all chromosome arms in WT and *∆MED12* cells. Lines connect scores for the same chromosome arm. **C** Compartment scores for a specific region on chromosome 8 for WT (black) and mutant (blue) cells. Black rectangles show hypercompartmentalized domains (HCDs) identified by a custom hidden markov model. **D** Venn diagram shows the overlap in genome-wide coverage between HCDs identified in *∆MED12* and H3K9me3 domains identified in WT cells. **E** TAD borders in HCDs were stratified into three categories depending on their difference in TAD separation (“weaker”, “unchanged”, “stronger”). The relative position within an HCD is plotted. **F** ICE normalized Hi-C contact matrix at 20 kb resolution for WT and *∆MED12* cells are shown for a region on chromosome 21. Contact matrices are visualized using GENOVA. Black rectangles indicate the position of HCD.

MED12 has been suggested to be involved in the bridging of promoters to enhancers^22,23^, although this role of Mediator has been questioned in two recent reports^24,25^. To understand the role of MED12 in 3D genome organization in our system, we generated high-resolution in situ Hi-C maps for *∆MED12* cells. When we zoom in to a single chromosome arm, we see that there is a clear increase in compartmentalization in *∆MED12* compared to wild-type cells (Fig. 3A, Supplementary Fig. 4a). By quantifying the degree of compartmentalization (i.e. compartment strength^26^), we find that compartmentalization increases for almost every chromosome arm (Fig. 3B, Supplementary Fig. 4b). Upon studying the Hi-C matrices, we noted that the compartmentalization seemed to increase only for the B compartment. Comparison of wild-type and *∆MED12* cells overall indeed showed stronger compartment scores for the B compartment, whereas regions in the A-compartment remained largely unchanged (Fig. 3C). We devised a hidden Markov model to identify regions of increased compartmentalization. We refer to these regions as hypercompartmentalized domains (HCDs), which together cover 633 Mb (22.1% of the genome). As expected, HCDs showed considerable overlap with H3K9me3 domains (Fig. 3D). Collectively, these results would suggest that the Mediator-CDK-module somehow restricts heterochromatinization and prevents over-compartmentalization.

Compartments can be subdivided into submegabase-sized topologically associating domains (TADs). These are formed by cohesin-mediated loop extrusion^10,12,27^. We analyzed what happens to TAD borders (i.e. genomic regions separating two TADs) that are located in HCDs. We find that TAD borders that become stronger (i.e. showing stronger segregation) are located closer to the boundaries of HCDs (Fig. 3E), consistent with the observed increase in compartmentalization. Conversely, TAD boundaries that decrease in strength are found closer to the center of HCDs (Fig. 3E). This suggests that TAD-border separation, which is controlled by cohesin and CTCF, can be disrupted in *∆MED12* cells. Figure 3F shows an example of an HCD where there is a clear loss of a TAD border inside an HCD. This region also reveals a loss of CTCF-anchored chromatin loops within the region. Note that when we calculate the aggregate signal of all chromatin loops identified in wild-type cells, we do not see a clear difference (Supplementary Fig. 4c). Together these results indicate that MED12 deficient cells display less clear TAD boundaries and a loss of CTCF-anchored loops, but that both these phenotypes are particularly pronounced within heterochromatin domains.

**Fig. 4:**
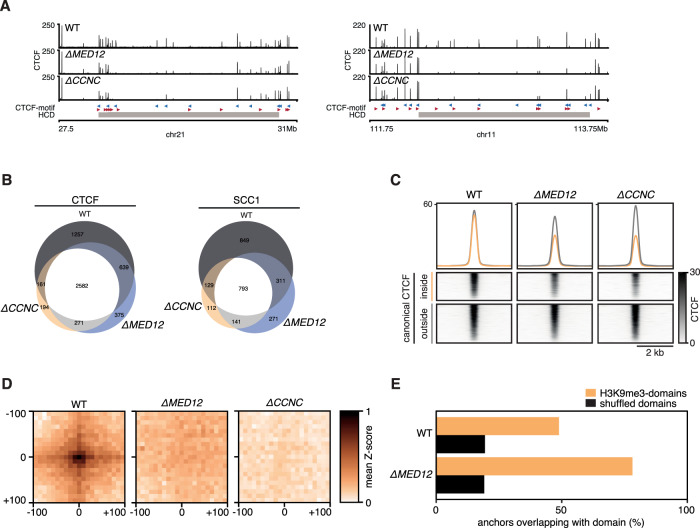
Mediator-CDK subunits control canonical CTCF-cohesin binding sites. **A** Two example regions showing CTCF binding in WT, *∆MED12* and *∆CCNC* cells. Gray rectangle indicates position of HCD **B** Venn diagram showing the overlap between CTCF and SCC1 peaks in WT, *∆MED12* and *∆CCNC* cells. **C** Aggregate alignment of CTCF ChIPseq signal on canonical (i.e. CTCF/SCC1 co-bound sites) stratified for inside and outside a H3K9me3 domain. **D** Virtual loop anchorpoints were selected as intersection of CTCF and SCC1 sites that were in the top 10% with the strongest decrease in signal. Pairwise alignment of Hi-C signal (PE-SCAn) on virtual anchorpoints. **E** Relative enrichment of lost virtual anchorpoints inside H3K9Me3 domains versus expected based on circular permutation (shuffled).

To better understand the role of the CDK-module in 3D genome organization, we mapped the binding sites of the architectural proteins CTCF and SCC1 (also known as RAD21, a subunit of the cohesin complex) using ChIPseq in wild-type, *∆MED12* and *∆CCNC* cells. The binding sites that were identified in wild-type were comparable to our previously published ChIPseq tracks for these factors (Supplementary Fig. 4e). Inspection of the binding tracks revealed that CTCF binding was diminished at numerous sites in HCDs (Fig. 4A). The identified binding sites in HCDs of *∆MED12* and *∆CCNC* cells decreased in number compared to wild-type in HCDs, as well as in signal strength (Fig. 4B, C), while CTCF protein levels were unaffected (Supplementary Fig. 4d). To link these changes to genome organization, we selected the CTCF/SCC1 binding sites that show the greatest relative decline in binding signal for CTCF and SCC1. By performing a pile-up of Hi-C signal on pairwise combinations of these sites, we find that wild-type cells display chromatin loops at these sites, and that these loops are ablated in *∆MED12* and *∆CCNC* cells (Fig. 4D). The CTCF/SCC1 sites with the strongest decline are heavily skewed towards H3K9me3 domains (Fig. 4E). Notably, the vast majority of chromatin loops identified using HICCUPs^28^ in wild-type do not colocalize with MED12-peaks (Supplementary Fig. 4F).

**Fig. 5:**
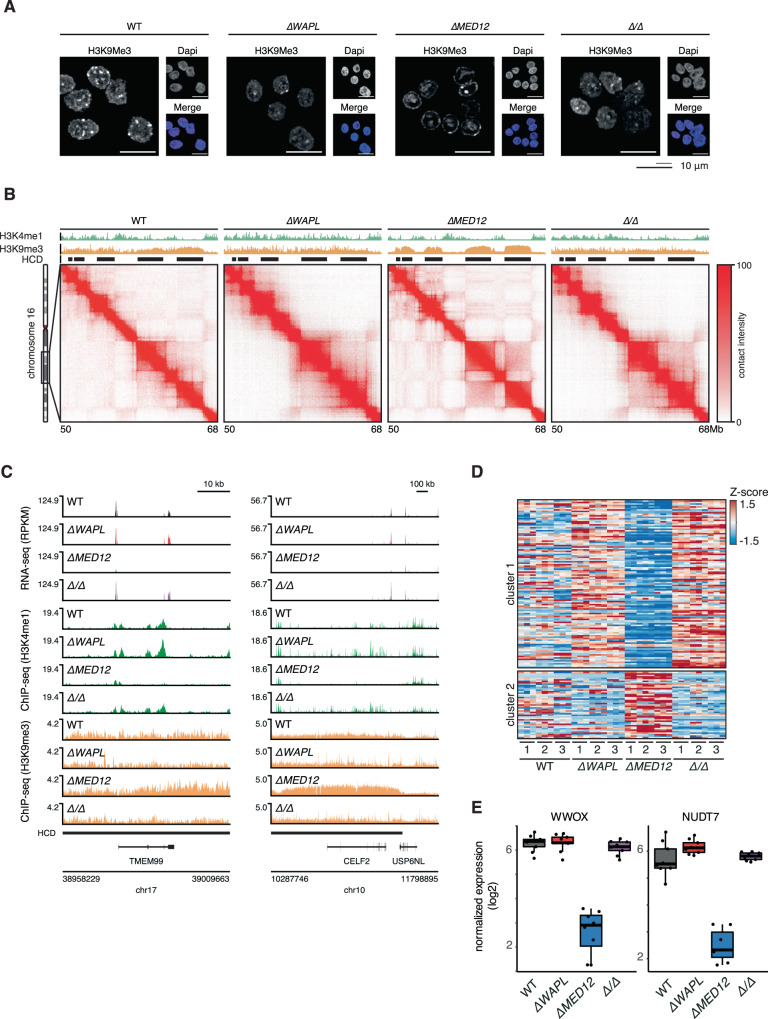
MED12 maintains a transcription-permissible 3D genome organization. **A** Immunofluorescence analysis of H3K9me3 levels in WT, *∆WAPL*, *∆MED12* and *∆WAPL/∆MED12* (*∆/∆*) cells. Representative images are shown from experiments that have been performed twice (see Supplementary Fig. 5b). **B** ICE normalized Hi-C contact matrix at 20 kb resolution for WT, *∆MED12*, *∆WAPL* and *∆WAPL/∆MED12* cells are shown for a region on chromosome 16. Contact matrices are visualized using GENOVA. Black rectangles indicate the position of HCDs. **C** RNA-seq and ChIP-seq profiles of TMEM99 (left) and CELF2 (right) in WT, *∆MED12*, *∆WAPL* and *∆WAPL/∆MED12* cells. Triplicate datasets are overlayed per cell line. **D** RNAseq heatmap showing expression of genes in H3K9me3 domains. K-means clustering (*k* = 2) reveals a cluster of silenced genes in H3K9me3. **E** Boxplots show expression of two example genes on chromosome 16. Boxplots show the expression levels in the RNAseq experiments (*n* = 9) for all assayed genotypes. Boxes indicate the interquartile range (IQR) of the data (25–75%) and box center line indicates the median. Whiskers extend to the minimum or maximum value that lies no further than 1.5 times the IQR from the bottom or top of the box.

Our genetic screening data showed that mutations in CDK-module subunits were better tolerated in *∆WAPL* cells. We therefore mutated *MED12* in a *∆WAPL* background to create double knock-out lines. When we perform immunofluorescence analysis of H3K9me3, we find that the pronounced heterochromatin domains of the *∆MED12* cells are absent in the *∆WAPL/∆MED12* cells (Fig. 5A, Supplementary Fig. 5b). Consistent with the immunofluorescence results, we find by ChIPseq that H3K9me3 domains are strongly diminished (Fig. 5B, Supplementary Fig. 5c–f), and by Hi-C we find that the hyper-compartmentalization seen in *∆MED12* is also completely rescued. These results indicate that the stabilization of chromatin loops by WAPL-ablation can counteract the increased compartmentalization and heterochromatinization that is conferred by loss of *MED12*.

An important remaining question is how these changes in the 3D genome and epigenome affect gene expression. To this end, we performed RNA-seq analysis on the wild-type, *∆WAPL*, *∆MED12,* and *∆WAPL/∆MED12* cell lines. When we analyze the genes located in H3K9me3 domains, we find a large cluster of genes whose expression is downregulated in *∆MED12* cells, consistent with the increased heterochromatinization (Fig. 5C–E). However, when MED12 is knocked out in the *∆WAPL* background, these genes are not downregulated. These results show that these genes are not dependent on MED12 per se for their expression, and would suggest that the 3D genome organization rather dictates the expression of these genes. MED12 therefore may contribute to the maintenance of a 3D genome organization that is permissible to gene expression.

Our functional analyses show that the 3D genome is intimately linked to the epigenome and gene expression. How then does loss of the Mediator-CDK-module subunits lead to such major changes in the epigenome, and in particular to the amplification of heterochromatin domains? Our analyses show that loss of this module results in reduced CTCF and cohesin binding, and correspondingly in a loss of chromatin loops. Conversely, we find that stabilization of such loops by the loss of WAPL results in the near-ablation of heterochromatin domains. We therefore suggest that chromatin loops counteract the formation of heterochromatin domains.

**Fig. 6:**
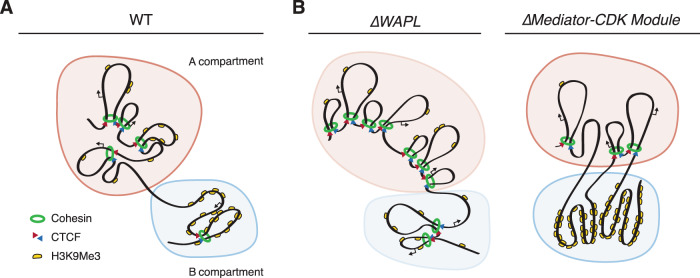
The Mediator CDK Module and cohesin play distinct and opposite roles in the genomic distribution of heterochromatin. **A** Heterochromatin domains, marked by H3K9me3, cluster together in three-dimensional space, separating the genome in A- and B compartments. **B** Model for how cohesin and Mediator-CDK may play opposite roles in heterochromatin domain formation. Upon loss of the cohesin-release factor WAPL and stabilization of cohesin, loops increase in size, leading to a loss of compartmentalization. This perturbs the formation and spreading of heterochromatin domains. Loss of members of the Mediator-CDK-Module results in unconstrained heterochromatin-domain amplification and hyper-compartmentalization, impairing cohesin- and CTCF binding and a loss of associated loops.

An explanation for the over-heterochromatinization upon the loss of loops may lie in the microphase separation model that has been proposed for the organization of the 3D genome into compartments^29^. Heterochromatin has the characteristics of a phase-separated compartment^3^ and its constituent protein HP1 can phase-separated in vitro^30^. We propose that chromatin loops counteract the phase separation of heterochromatin components. On one hand, WAPL, by driving cohesin’s release from DNA, allows the formation of heterochromatin domains. On the other hand, a fully intact Mediator-CDK-module counteracts the formation of heterochromatin domains (Fig. 6). It should be noted that in our analyses we have used clonal knock-out lines of MED12 and CCNC and therefore we cannot exclude that the observed effects occur through an, as of yet unidentified, indirect mechanism. Given the crucial role of the Mediator complex in gene expression, we cannot rule out that a regulator of heterochromatin is misexpressed. We have scrutinized our RNAseq data for indications that epigenetic modifiers are changed in gene expression, and found no obvious candidate. However, we cannot exclude the possibility of changes in the expression of an unknown regulator of heterochromatin domains.

While we cannot rule out an indirect regulatory mechanism, the Mediator-CDK-module may in fact be directly involved in regulating heterochromatin domains. Below we discuss two, not necessarily mutually exclusive mechanisms, for how the Mediator-CDK-module might restrain heterochromatin domain formation. One possibility is through the direct action of the Mediator-CDK-subunit CDK8, which has been shown to be a histone H3 kinase^31^. Phosphorylation of S10 of H3 blocks the binding of HP1 to H3K9me3^32,33^. Loss of MED12 may lead to a decrease in the activity of the module and hence a decrease in H3S10P, which in turn could allow for increased heterochromatin domain formation. Alternatively, MED12 may play a role in the formation and/or stabilization of loops, as has been suggested previously^22,34^. Loss of MED12 could hereby lead to enhanced phase-separation of heterochromatin domains, leading to unrestrained amplification of dense heterochromatin domains.

This would also explain the loss of heterochromatin domains in the absence of WAPL: a stabilization of loops would then counteract the phase-separation of heterochromatin domains, and hereby prevent their self-amplification. Indeed, high-resolution DNA FISH experiments have shown that loss of WAPL results in increased interactions between neighboring domains^35^, consistent with our Hi-C data. This domain intermingling, particularly between heterochromatic (H3K9me3 high) and euchromatic (H3K9me3 low) domains may explain the redistribution of this epigenetic mark. An increase in heterochromatin density, as observed in the Mediator-CDK-module mutants, could in turn limit the binding of CTCF and cohesin, which then could allow for yet further heterochromatinization. The absence of a counterforce could therefore result in a shift in the epigenome, in which all heterochromatin that would normally reside at dispersed focal H3K9me3 peaks now ends up in monolithic heterochromatin domains. We propose that heterochromatin domains could thus act as a sink for heterochromatin components. By preventing the formation of such dense heterochromatin structures, MED12 might contribute to the maintenance of a 3D genome organization that is permissible to gene expression. As such we have uncovered a previously unappreciated link between nuclear organization and the epigenome, and we suggest that this inter-connected network could control gene regulation in an unexpected way.

## Methods

### Genome editing and cell culture

Hap1 cells were cultured in Iscove’s Modified Dulbecco’s Medium (IMDM) supplemented with 10% FCS (Clontech), 1% Penicillin/Streptomycin (Invitrogen) and 0.5% UltraGlutamin (Lonza). Hap1 knock-out clones were generated using gRNA’s targeting MED12 (primer: 5′CCCTTCCTGACACTGGTCGC-3′) and (primer: 5′GATTGCTGCATAGTAGGCAC-3′) and targeting CCNC (5′TACTGGAGCAAAGGTCTATA-3′), which were cloned into PX330. HAP1 wild-type or *∆WAPL* cells^9^, were transfected with PX330 and pDonorTia containing a puromycin or blasticidin resistance gene^17^. Clones were selected using puromycin (2 µg/µl) or blasticidin (10 ug/ul). Colonies were screened for the loss of MED12 using PCR and western blot analysis. For MED12 the following primers were used: 5′TGGCTGGGAATCCTAGTGAC-3′, 5′ACCGAGCTAGGCTATGGGAA-3′, 3′TTTGGCTAGTTGCGTGAGTG-5′, 3′CGTGAGGAAGAGTTCTTGCAG-5′ and 3′AGGCCTTCCATCTGTTGCT-5′, for CCNC: 5′GGGAATGAATGGATGCTGAC-3′, 3′ACATCAAGGCAAAAGGATGG-5′ and 3′GACATGGTGCTTGTTGTCCTC-5′.

### Hi-C

We performed in-nucleus or in situ Hi-C^36–38^ as described in ref. ^9^. Hi-C libraries were sequenced on a HiSeq X. Hi-C sequencing data was processed with Hi-C-Pro 2.9^39^. We performed loop calling with HICCUPs 0.9^28^. Further data analysis was performed with GENOVA^40^ (github.com/deWitLab/GENOVA), see below for details.

### ChIPseq & RNAseq

Samples for RNAseq were prepared as previously described^9^. To ensure biological reproducibility, we analyzed different genetic clones for every knock-out line, and multiple replicates per clone. For ChIPseq samples were crosslinked for 10 min in 1% formaldehyde. For cohesin and CTCF ChIPs we used 15 milion cells and for the histone modifications 3 million cells. Crosslinking was stopped by adding Glycin (0.1 M end concentration). Cells were washed twice with PBS by centrifugation for 5 min at 1350 G. The cells were lysed in 10 ml lysis buffer 1 (50 mM HEPES pH7.5, 140 mM NaCl, 1 mM EDTA, 10% Glycerol, 0.5% NP40, 0.25% Triton X-100) at 4 °C. Cells were centrifuged for 5 min at 1350 G and the cell pellet was incubated for 10 min in lysis buffer 2 (200 mM NaCl, 1 mM EDTA, 0.5 mM EGTA, 10 mM Tris-HCl). After centrifugation the pellet was resuspended in lysis buffer 3 (100 mM NaCl, 1 mM EDTA, 0.5 mM EGTA, 10 mM Tris-HCl ph8, 0.1% DOC, 0.5% N-Lauroyl sarcosine). To shear the DNA we used for the histone modifications a covaris ultrasonicator in covaris microtubes AFA (ref 520045) under the following conditions: peak power 75 W, duty cycle 20%, cycle/burst 200 for 6 min. For cohesin and CTCF we used a Bioruptor Pico (Diagenode), with 5 cycles 15 s on, 90 s off. Magnetic beads (Dynabeads) were coupled to following antibodies, SCC1 (ab992, Abcam), CTCF (3418 S, Cell Signaling), H3K4me3 (PAB-003-050, Diagenode), H3K4me1 (PAB-037-050, Diagenode), H3K36me3 (MAB-183-050, Diagenode), H3K27me3 (PAB-195-050, Diagenode), H3K9me3 (PAB-193-050, Diagenode). For SCC1, CTCF, H3K4me3, H3K4me1, H3K36me3, H3K27me3, and H3K9me3 we used 5 μg of antibody plus 50 μl of beads. Whole cell extract was added to the beads and incubated overnight. IP’s were washed at least 4 times with RIPA buffer (50 mM HEPES pH7.5, 1 mM EDTA, 0.7% DOC, 1% NP-40, 0.5 M LiCl) and once with cold TE, 50 mM NaCl. Material was eluted overnight with 150 µl elution buffer (50 mM Tris-HCl pH8, 10 mM EDTA, 1% SDS) at 65 °C. The DNA was recovered with the Qiaquick pcr purification kit (Qiagen 28106). For the MED12 ChIP we used 7 μg of antibody (A300-774A Bethyl) plus 70 μl of beads. Cells were fixed on the plate with 2 mM disuccinimidyl glutarate (DSG) for 1 h with 1% formaldehyde being added the last 15 min. Libraries for CTCF, SCC1, MED12 and histone modification ChIPs were prepared with the KAPA HTP library preparation kit (KK8234 Illumina) and subsequently sequenced on a HiSeq 2500 (single-end).

### Hi-C analysis

The compartment score was calculated as described in refs. ^11,41^. Briefly, per chromosome arm we calculate an observed over expected (O/E) matrix with a resolution of 100 kb. We subtract 1 from the O/E matrix and extract the eigen vectors. The first eigen vector is multiplied by the square root of the eigen value of this vector resulting in the compartment score. As the compartment score is a dimensionless score, we use the H3K4me1 signal to correctly orient the compartment score. To calculate the compartment strength we selected the bins with top 20% and bottom 20% of the compartment score as A and B compartments, respectively. The compartment strength is then calculated as the log2 of product of the interactions between two A compartments (AA) and two B compartments (BB) divided by the square of the interactions between an A and a B compartment (AB): log_2_ (AAxBB)/AB^2^.

To identify hyper-compartmentalized domains, a hidden Markov model (HMM) was used. The model was specified as having two hidden states, stable vs. differential compartment score, and two gaussian responses: the *∆MED12* compartment scores and the absolute difference in compartment scores between wild-type and *∆MED12* on the 40 kb resolution matrices. Parameters were optimized by the default EM algorithm of depmixS4^42^. This model was used to classify states on all chromosome arms separately with 40 kb resolution compartment scores as input. Afterwards, two filtering steps were applied to call HCDs: (i) we selected only regions with negative compartment scores in *∆MED12* Hi-C data and (ii) only regions with five consecutive differential states were called as HCDs.

Insulation scores were calculated as described in ref. ^43^. We compared the insulation score of TAD borders called in wild-type^9^ for wild-type and *∆MED12* lines and ordered the borders on the difference in the insulation score. We stratified the TAD borders located in an HCD into three equal-sized categories based on the difference in insulation score: weakened borders (higher insulation score), stronger borders (lower insulation score) and equal border (little to no difference in insulation score). We determined the position of the TAD borders in all three categories with respect to the edges of HCDs.

### ChIPseq data analysis

Mapping of ChIPseq data was performed with bowtie 2.3.4.1^44^ to hg19. We performed peak calling with MACS2 2.1.1^45^ for SCC1, CTCF, H3K4me3, H3K4me1, and H3K36me3 with standard settings. For H3K9me3 we performed peak calling in advanced mode using the following settings -l 500 -g 65 -cutoff-analysis 2.25. ChromHMM was performed as described in ref. ^21^. H3K9me3 domains were also called with MACS2 in advanced mode using the following settings -l 500 -g 65 -G 2000. Where indicated we filtered out H3K9me3 peaks that overlapped with H3K9me3 domains. ChIPseq alignment plots were created with deeptools 3.0.0.

### RNAseq data analysis

RNAseq data were mapped with TopHat 2.1.1^46^ and count-tables were generated with HTseq^47^ with the stranded=reverse setting using the Gencode v27lift37 gene-build. TPMs were calculated with DESeq2 1.18.1^48^ by dividing the counts by the normalization factors. Differential genes were called using DESeq2 using the Wald test. Profiles were generated with RPKM-normalized tracks in triplicate. Our RNAseq data offered the opportunity to check whether the changes in 3D genome organization and chromatin landscape were due to a change in the RNA levels of obvious chromatin regulatory or architectural proteins. No obvious genes or overarching functional categories were found enriched among up- or downregulated genes (Supplementary Fig. 3b, c). Furthermore, the expression of *CTCF*, *RAD21*, HP1-isoforms, and DNA-methyltransferases and -demethylases was unaltered (Supplementary Fig. 3a). We selected genes that overlapped with wild-type H3K9me3 domains. We performed k-means clustering on the genes with k = 2.

### Immunofluorescence

Cells were grown on poly-l-lysine (Sigma-Aldrich) coated coverslips and samples were taken at 30% confluency. Non-chromatin bound proteins were removed using a pre-extraction procedure of 0.1% Triton X-100 in PBS for 1 min, followed by fixation using 3.7% formaldehyde in PBS for 7 min. Samples were blocked using 4% BSA in PBS for 1 h at room temperature and incubated with primary H3K9Me3 (ab8898, Abcam) or HP1alpha (Clone 15 19s2, Upstate/MilliporeSigma) antibody at a 1:1000 dilution overnight at 4 °C. Samples were washed (0.1% Tween in PBS) and incubated using secondary antibody (either goat anti-Mouse Alexafluor 488 or 568 (ThermoFisher A-11029 and A-11004), and goat anti-Rabbit Alexafluor 488 or 568 (ThermoFisher A-11008 and A-11011), used at a 1:600 dilution) and DAPI (Sigma-Aldrich). Coverslips were mounted onto glass slides using Prolong Antifade Gold (Invitrogen). Representative images were acquired using a Leica Confocal microscope 63x/1.32 oil lens using LAS-AF Software (Leica), DAPI levels were adjusted for visualization purposes. Analysis of the intensity of H3K9me3 or HP1 in the DAPI region was performed using an in-house written macro (ImageJ).

### Western blot

The following antibodies were used for western blots: WAPL (A-7, sc-365189, Santa Cruz), HSP90 (F-8, sc-13119, Santa Cruz), MED12 (A300-774A, Bethyl), CCNC (ab85927, Abcam), CTCF (ab70303, Abcam), Actin (ab6276, Abcam) and Tubulin (T5168, Sigma-Aldrich). All primary antibodies were used at a 1:1000 dilution, except for Tubulin 1:4000. Secondary antibodies for western blot analysis were used in a 1:2000 dilution: Goat anti-Rabbit-PO and Goat anti-Mouse-PO (DAKO P0448 and P0447, respectively).

### TLA

TLA was performed as previously described^18^, using the default restriction enzyme combination (i.e NlaIII as a first restriction enzyme and NspI as the second). We designed TLA primers that on the resistance marker sequence (i.e. Blasticidin or Puromycin) to amplify from the inserted cassette. Blasticidin primers were: forward: GCTAGTTCAAACCTTGGGAAAA, reverse: ATGAGCACAAAGCAGTCAGG. Puromycin primers were: forward: GCAACCTCCCCTTCTACGAG, reverse: AGGCCTTCCATCTGTTGCT. Sequencing libraries were generated using in-house produced Tn5 enzyme^49^ and sequenced on HiSeq 2500 single end 65 nt.

### Reporting summary

Further information on research design is available in the Nature Research Reporting Summary linked to this article.

## Supplementary information

Supplementary Information

Reporting Summary

## Data Availability

The data that support this study are available from the corresponding authors upon reasonable request. The RNAseq, ChIPseq and Hi-C data generated in this study have been deposited in the NCBI GEO database under accession code GSE125672. The public datasets used in this study are available in the GEO database under accession code GSE95015. Source data are provided with this paper.

## Source data

Source Data

## Code availability

Code used for sequencing data analysis and image analysis scripts have been deposited at: https://github.com/deWitLab/Haarhuis_2022_NatureCommunications [https://github.com/deWitLab/Haarhuis_2022_NatureCommunications]. Hi-C data has been analysed with GENOVA which can be downloaded here: https://github.com/deWitLab/GENOVA [https://github.com/deWitLab/GENOVA].

## References

[1] Rea S (2000). Regulation of chromatin structure by site-specific histone H3 methyltransferases. Nature.

[2] Elgin, S. C. R. & Reuter, G. Position-effect variegation, heterochromatin formation, and gene silencing in Drosophila. *Cold Spring Harb. Perspect. Biol.* 5, a017780 (2013). 10.1101/cshperspect.a017780 PMC3721279 23906716

[3] Strom AR (2017). Phase separation drives heterochromatin domain formation. Nature.

[4] Wang J, Jia ST, Jia S (2016). New insights into the regulation of heterochromatin. Trends Genet..

[5] Ryba T (2010). Evolutionarily conserved replication timing profiles predict long-range chromatin interactions and distinguish closely related cell types. Genome Res..

[6] Guelen L (2008). Domain organization of human chromosomes revealed by mapping of nuclear lamina interactions. Nature.

[7] Lieberman-Aiden E (2009). Comprehensive mapping of long-range interactions reveals folding principles of the human genome. Science.

[8] Stevens, T. J. et al. 3D structures of individual mammalian genomes studied by single-cell Hi-C. *Nature*10.1038/nature21429 (2017). 10.1038/nature21429 PMC5385134 28289288

[9] Haarhuis JHI (2017). The cohesin release factor WAPL restricts chromatin loop extension. Cell.

[10] Wutz, G. et al. Topologically associating domains and chromatin loops depend on cohesin and are regulated by CTCF, WAPL, and PDS5 proteins. *EMBO J.* e201798004 10.15252/embj.201798004 (2017). 10.15252/embj.201798004 PMC5730888 29217591

[11] Schwarzer, W. et al. Two independent modes of chromatin organization revealed by cohesin removal. *Nature*10.1038/nature24281 (2017). 10.1038/nature24281 PMC5687303 29094699

[12] Rao SSP (2017). Cohesin loss eliminates all loop domains. Cell.

[13] Li, Y. et al. The structural basis for cohesin-CTCF anchored loops. *Nature* **578**, 472–476. 10.1038/s41586-019-1910-z PMC7035113 31905366

[14] Despang A (2019). Functional dissection of the Sox9–Kcnj2 locus identifies nonessential and instructive roles of TAD architecture. Nat. Genet..

[15] Flavahan WA (2019). Altered chromosomal topology drives oncogenic programs in SDH-deficient GISTs. Nature.

[16] Allahyar A (2018). Enhancer hubs and loop collisions identified from single-allele topologies. Nat. Genet..

[17] Blomen VA (2015). Gene essentiality and synthetic lethality in haploid human cells. Science.

[18] de Vree, P. J. P. et al. Targeted sequencing by proximity ligation for comprehensive variant detection and local haplotyping. *Nat. Biotechnol.*10.1038/nbt.2959 (2014). 10.1038/nbt.2959 25129690

[19] Fant, C. B. & Taatjes, D. J. Regulatory functions of the Mediator kinases CDK8 and CDK19. *Transcription* 1–15. 10.1080/21541264.2018.1556915 (2018). 10.1080/21541264.2018.1556915 PMC6602567 30585107

[20] Kundaje A (2015). Integrative analysis of 111 reference human epigenomes. Nature.

[21] Ernst J, Kellis M (2012). ChromHMM: automating chromatin-state discovery and characterization. Nat. Methods.

[22] Kagey MH (2010). Mediator and cohesin connect gene expression and chromatin architecture. Nature.

[23] Phillips-Cremins JE (2013). Architectural protein subclasses shape 3D organization of genomes during lineage commitment. Cell.

[24] El Khattabi L (2019). A pliable mediator acts as a functional rather than an architectural bridge between promoters and enhancers. Cell.

[25] Jaeger, M. G. et al. Selective Mediator dependence of cell-type-specifying transcription. *Nat. Genet.*10.1038/s41588-020-0635-0 (2020). 10.1038/s41588-020-0635-0 PMC7610447 32483291

[26] Imakaev M (2012). Iterative correction of Hi-C data reveals hallmarks of chromosome organization. Nat. Methods.

[27] Fudenberg, G. et al. Formation of chromosomal domains by loop extrusion. *Cell Rep.*10.1016/j.celrep.2016.04.085 (2016). 10.1016/j.celrep.2016.04.085 PMC4889513 27210764

[28] Durand NC (2016). Juicer provides a one-click system for analyzing loop-resolution Hi-C experiments. Cell Syst..

[29] Falk, M. et al. Heterochromatin drives compartmentalization of inverted and conventional nuclei. *Nature*10.1038/s41586-019-1275-3 (2016). 10.1038/s41586-019-1275-3 PMC7206897 31168090

[30] Larson AG (2017). Liquid droplet formation by HP1α suggests a role for phase separation in heterochromatin. Nature.

[31] Knuesel, M. T., Meyer, K. D., Donner, A. J., Espinosa, J. M. & Taatjes, D. J. The human CDK8 subcomplex is a histone kinase that requires Med12 for activity and can function independently of mediator. *Mol. Cell. Biol.*10.1128/mcb.00993-08 (2009). 10.1128/MCB.00993-08 PMC2630685 19047373

[32] Fischle W (2005). Regulation of HP1–chromatin binding by histone H3 methylation and phosphorylation. Nature.

[33] Hirota T, Lipp JJ, Toh B-H, Peters J-M (2005). Histone H3 serine 10 phosphorylation by Aurora B causes HP1 dissociation from heterochromatin. Nature.

[34] Lai, F. et al. Activating RNAs associate with Mediator to enhance chromatin architecture and transcription. *Nature*10.1038/nature11884 (2009). 10.1038/nature11884 PMC4109059 23417068

[35] Luppino, J. M. et al. Cohesin promotes stochastic domain intermingling to ensure proper regulation of boundary-proximal genes. *Nat. Genet.*10.1038/s41588-020-0647-9 (2020). 10.1038/s41588-020-0647-9 PMC7416539 32572210

[36] Splinter E, de Wit E, van de Werken HJG, Klous P, de Laat W (2012). Determining long-range chromatin interactions for selected genomic sites using 4C-seq technology: From fixation to computation. Methods.

[37] Rao SSPSP (2014). A 3D map of the human genome at kilobase resolution reveals principles of chromatin looping. Cell.

[38] Nagano T (2013). Single-cell Hi-C reveals cell-to-cell variability in chromosome structure. Nature.

[39] Servant N (2015). HiC-Pro: an optimized and flexible pipeline for Hi-C data processing. Genome Biol..

[40] van der Weide, R. H. et al. Hi-C analyses with GENOVA: a case study with cohesin variants. *NAR Genomics Bioinform*. 10.1093/nargab/lqab040 (2021). 10.1093/nargab/lqab040 PMC8140737 34046591

[41] Flyamer IM (2017). Single-nucleus Hi-C reveals unique chromatin reorganization at oocyte-to-zygote transition. Nature.

[42] Visser I, Speekenbrink M (2010). depmixS4: an R package for hidden Markov models. J. Stat. Softw..

[43] Crane E (2015). Condensin-driven remodelling of X chromosome topology during dosage compensation. Nature.

[44] Langmead B, Salzberg SL (2012). Fast gapped-read alignment with Bowtie 2. Nat. Methods.

[45] Zhang Y (2008). Model-based analysis of ChIP-Seq (MACS). Genome Biol..

[46] Kim D (2013). TopHat2: accurate alignment of transcriptomes in the presence of insertions, deletions and gene fusions. Genome Biol..

[47] Anders S, Pyl PT, Huber W (2015). HTSeq-a Python framework to work with high-throughput sequencing data. Bioinformatics.

[48] Love MI, Huber W, Anders S (2014). Moderated estimation of fold change and dispersion for RNA-seq data with DESeq2. Genome Biol..

[49] Picelli S (2014). Tn5 transposase and tagmentation procedures for massively scaled sequencing projects. Genome Res..

