# Supplementary material for: A Mediator-cohesin axis controls heterochromatin domain formation
Source: Nat Commun. 2022 Feb 8;13:754. doi: 10.1038/s41467-022-28377-7 (PMC8826356; doi:10.1038/s41467-022-28377-7)
Supplement: Supplementary file 1 — Supplementary Information [file 41467_2022_28377_MOESM1_ESM.pdf]

A Mediator-cohesin axis controls heterochromatin domain formation

Judith H.I. Haarhuis*, Robin H. van der Weide*, Vincent A. Blomen, Koen D. Flach, Hans Teunissen, Laureen Willems, Thijn R. Brummelkamp, Benjamin D. Rowland & Elzo de Wit

2022 Nature Communications

Supplementary Information

Supplementary Fig. 1: Characteristics of H3K9me3 ChIPseq data in mutant and WT HAP1 cells.

Supplementary Fig. 2: Validation of *ΔMED12* and *ΔCCNC* knock-outs.

Supplementary Fig. 3: Gene expression analyses in WT, *ΔWAPL*, *ΔMED12* and *Δ/Δ* cells.

Supplementary Fig. 4: Characterization of increased compartmentalization and binding characteristics of architectural proteins in *ΔMED12* and *ΔCCNC* cells.

Supplementary Fig. 5: Characterization of *ΔWAPL*/*ΔMED12* cells.

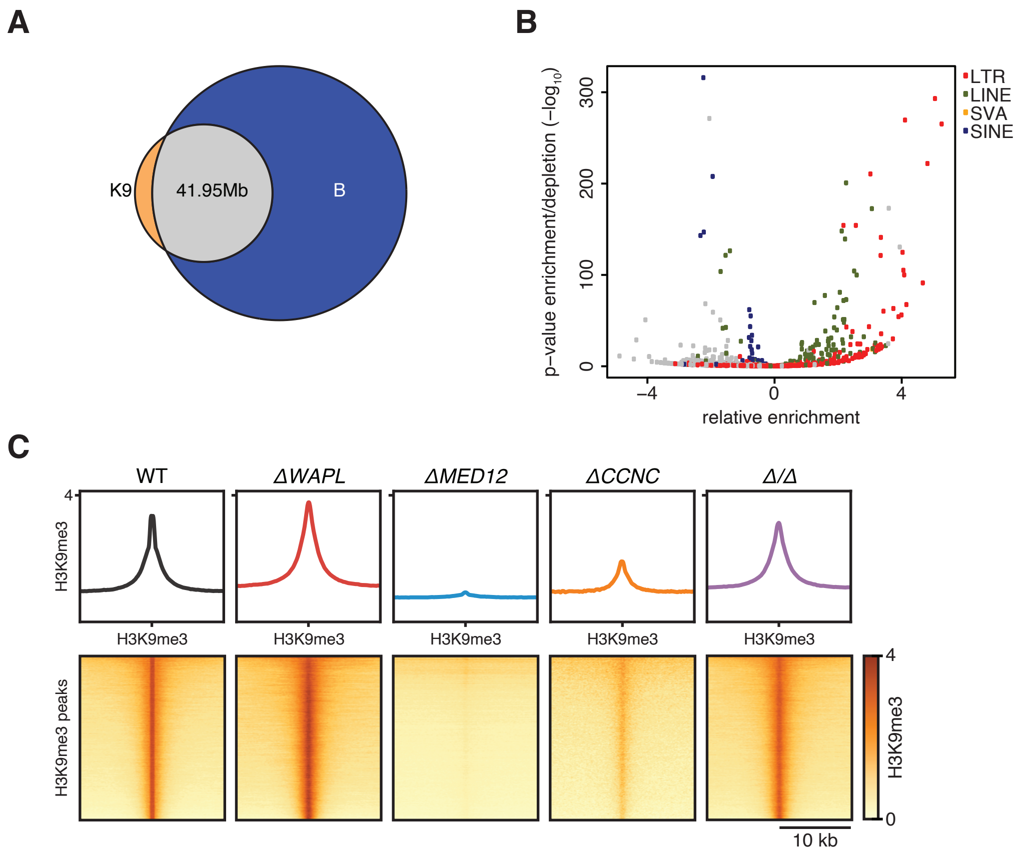

Supplementary Figure 1: Characteristics of H3K9me3 ChIPseq data in mutant and WT HAP1 cells. A) Venn diagram showing the overlap of WT H3K9me3 domains with WT B compartments from Hi-C data. B) Volcano plot showing the relative enrichment of H3K9me3 peaks in WT for specific transposable elements in A compartments. P-values are calculated using an upper or lower tailed binomial test depending on whether H3K9me3 peaks are enriched or depleted, respectively. C) Aggregate H3K9me3 ChIPseq signal over H3K9me3 peaks in A compartments for WT and mutant HAP1 cells, Δ/Δ is *ΔWAPL/ΔMED12* mutant.

!

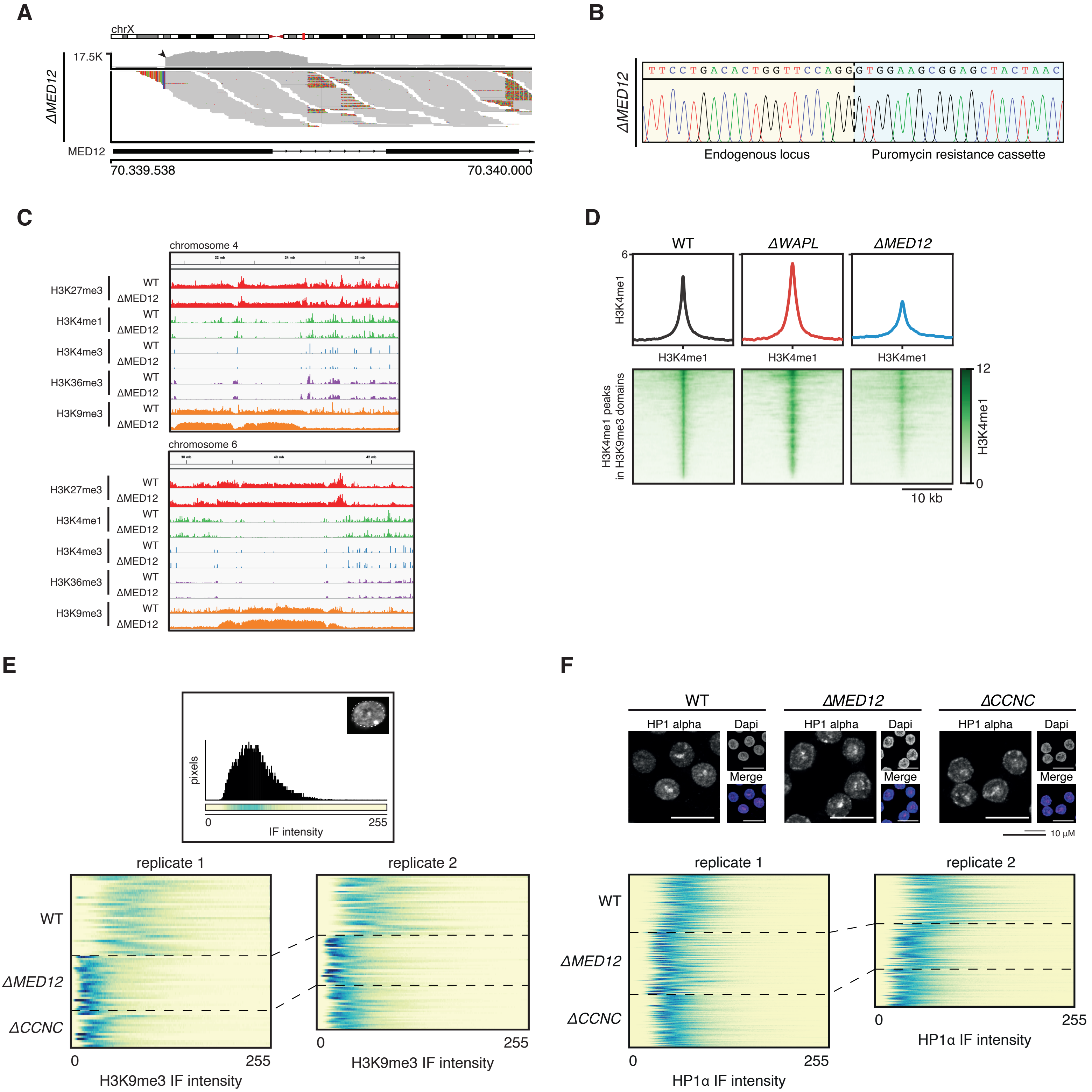

Supplementary Figure 2: Validation of *ΔMED12* and *ΔCCNC* knock-outs.

A) Targeted Locus Amplification (TLA) from the targeted gene trap construct reveals the nucleotide resolution integration site. B) Integration site is validated by Sanger sequencing. C) Example loci showing all five chromatin marks for WT and *ΔMED12*. D) Aggregate H3K4me1 ChIPseq signal over H3K4me1 peaks in WT, *ΔWAPL* and *ΔMED12* cells. E) Heatmaps showing the histogram for H3K9Me3 intensity for individual cells in WT, *ΔMED12* and *ΔCCNC*. F) Immunofluorescence analysis of HP1α levels in WT, *ΔMED12* and *ΔCCNC* cells (top). Representative images are shown from experiments that have been performed twice (see bottom panel). Heatmaps showing the histogram for HP1α intensity for individual cells in WT, *Δ MED12* and *ΔCCNC* (bottom).

!

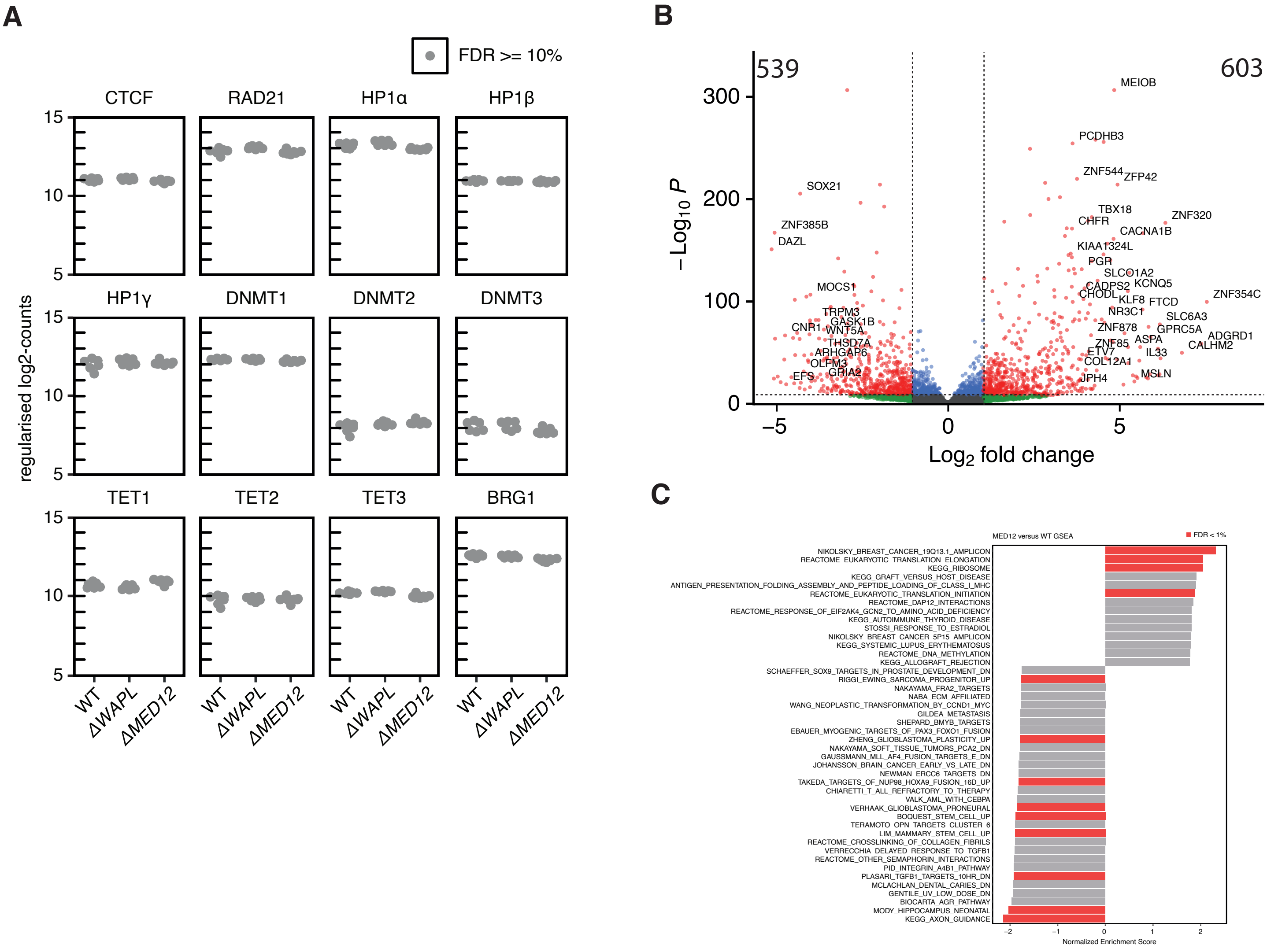

Supplementary Figure 3: Gene expression analyses in WT, *ΔWAPL*, *ΔMED12* and *Δ/Δ* cells.
A) Expression values of CTCF, RAD21, HP1-isoforms, DNA methyltransferases, DNA demethylases and BRG1. Every point denotes a replicate. FDR: false discovery rate. B) Volcano plot of RNAseq comparing WT and *ΔMED12* cells. Significant genes are highlighted in red and are identified using a two-sided Wald test implemented in DESeq2. C) Gene Set Enrichment Analysis (GSEA) of differentially expressed genes between WT and *ΔMED12* cells.
!

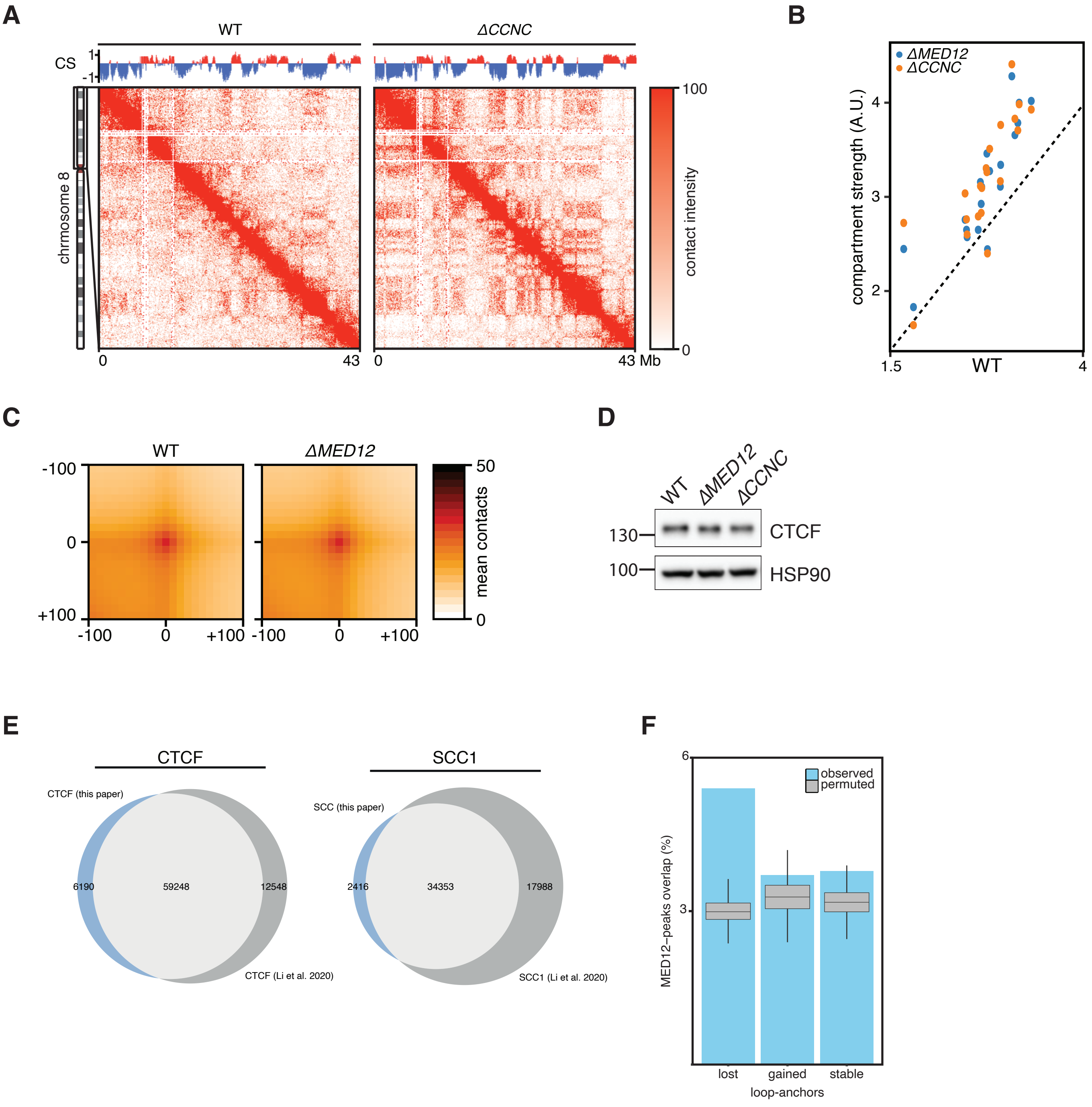

Supplementary Figure 4: Characterization of increased compartmentalization and binding characteristics of architectural proteins in *ΔMED12* and *ΔCCNC* cells.

A) ICE normalized Hi-C contact matrices at 100kb resolution for WT and *ΔCCNC* cells are shown for the p arm of chromosome 8. Above the matrices the compartment score is plotted. Contact matrices are visualized using GENOVA. B) Scatterplot shows the compartment strength for every chromosome in the *ΔMED12* and *ΔCCNC* cell lines compared to the WT data. Dotted line shows y=x, indicating no difference. C) Aggregate Peak Analysis (APA) of loops identified in WT cells in WT and *ΔMED12* cells. D) Western blot analysis of CTCF in wild-type and *ΔMED12*. Representative images are shown from experiments that have been performed twice. E) CTCF and cohesin WT ChIP-seq peaks in this paper compared to published datasets of Li et al. (2020). F) Barplot showing percentage of loop anchors containing MED12 ChIPseq peaks in loops detected in WT but not *ΔMED12* ('lost'), detected in *ΔMED12* but not WT ('gained') or detected in both ('stable'). Box and whiskers show the expected level based on circular permutation (n=1000). Boxes indicate the interquartile range (IQR) of the data (25%-75%) and box center line indicates the median. Whiskers extend to the minimum or maximum value that lies no further than 1.5 times the IQR from the bottom or top of the box, respectively.

!

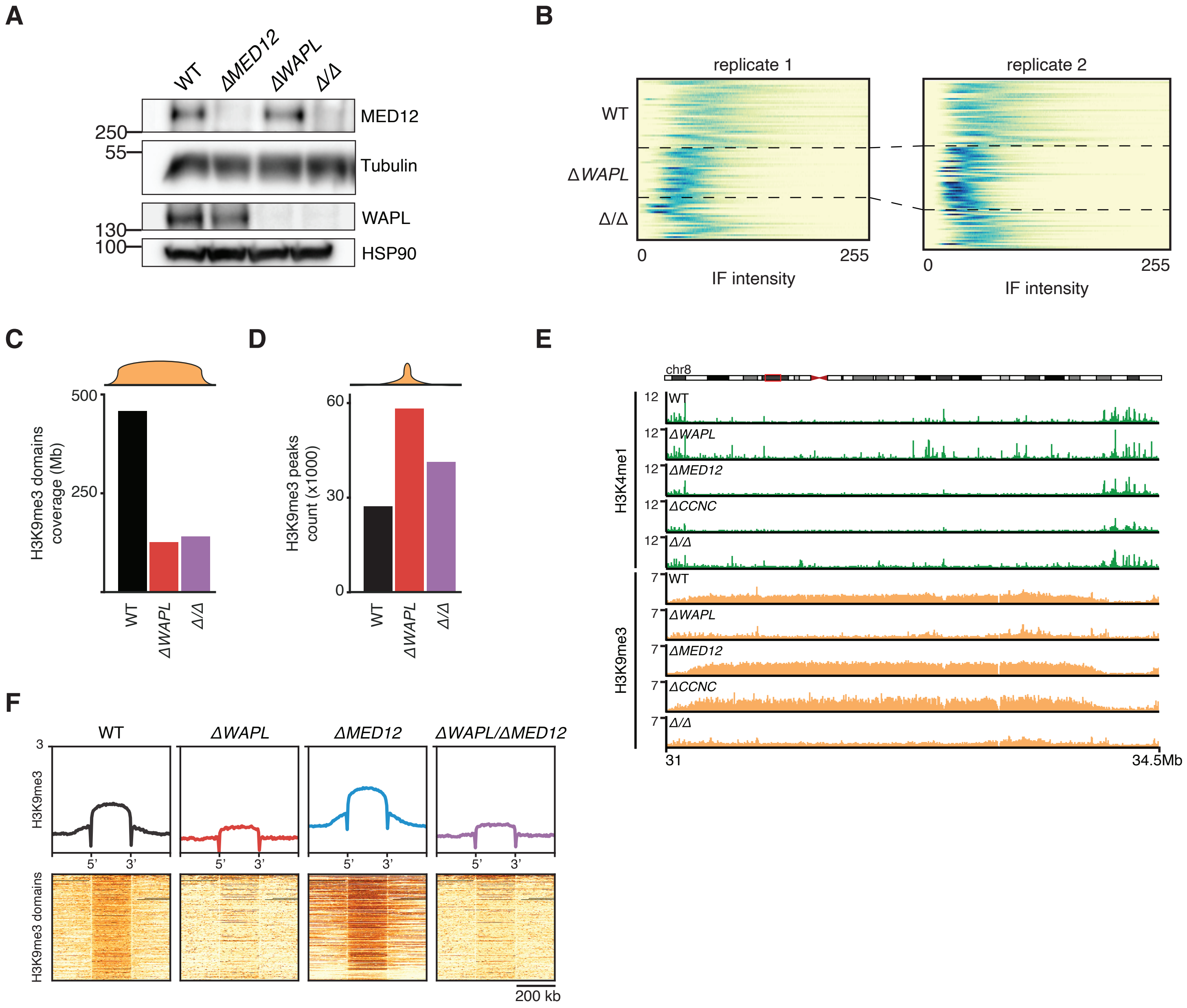

Supplementary Figure 5: Characterization of *ΔWAPL/ΔMED12* cells. A) Western blot confirming depletion of both MED12 and WAPL. HSP90 and Tubulin represent loading controls for MED12 and WAPL panels directly above. Representative images are shown from experiments that have been performed twice. B) Heatmaps showing the histogram for H3K9me3 intensity for individual cells in WT, *ΔWAPL* and *ΔWAPL/ΔMED12*. C) Genomic coverage of H3K9me3 domains in WT, *ΔWAPL* and *Δ WAPL/ΔMED12* cells. D) Number of H3K9me3 peaks in WT, *ΔWAPL* and *ΔWAPL/ΔMED12* cells. E) Example region showing the levels of H3K4me1 and H3K9me3 in WT, *ΔWAPL*, *ΔMED12*, *ΔCCNC* and *ΔWAPL/ΔMED12* cells. F) Aggregate analysis showing the H3K9me3 levels for all the WT H3K9me3 domains WT, *ΔWAPL*, *ΔMED12* and *ΔWAPL/ΔMED12* cells.

"
