# Supplementary material for: A Mediator-cohesin axis controls heterochromatin domain formation
Source: Nat Commun. 2022 Feb 8;13:754. doi: 10.1038/s41467-022-28377-7 (PMC8826356; doi:10.1038/s41467-022-28377-7)
Supplement: Supplementary file 2 — Reporting Summary [file 41467_2022_28377_MOESM2_ESM.pdf]

nature research

Corresponding author(s): Elzo de Wit, Benjamin Rowland

Last updated by author(s): Dec 23, 2021

Reporting Summary

Nature Research wishes to improve the reproducibility of the work that we publish. This form provides structure for consistency and transparency in reporting. For further information on Nature Research policies, see Authors & Referees and the Editorial Policy Checklist .

Statistics

For all statistical analyses, confirm that the following items are present in the figure legend, table legend, main text, or Methods section.

n/a	Confirmed	
☐	☒	The exact sample size (n) for each experimental group/condition, given as a discrete number and unit of measurement
☐	☒	A statement on whether measurements were taken from distinct samples or whether the same sample was measured repeatedly
☐	☒	The statistical test(s) used AND whether they are one- or two-sided<br>*Only common tests should be described solely by name; describe more complex techniques in the Methods section.*
☒	☐	A description of all covariates tested
☒	☐	A description of any assumptions or corrections, such as tests of normality and adjustment for multiple comparisons
☒	☐	A full description of the statistical parameters including central tendency (e.g. means) or other basic estimates (e.g. regression coefficient) AND variation (e.g. standard deviation) or associated estimates of uncertainty (e.g. confidence intervals)
☐	☒	For null hypothesis testing, the test statistic (e.g. F, t, r) with confidence intervals, effect sizes, degrees of freedom and P value noted<br>*Give P values as exact values whenever suitable.*
☒	☐	For Bayesian analysis, information on the choice of priors and Markov chain Monte Carlo settings
☒	☐	For hierarchical and complex designs, identification of the appropriate level for tests and full reporting of outcomes
☒	☐	Estimates of effect sizes (e.g. Cohen's d, Pearson's r), indicating how they were calculated

Our web collection on statistics for biologists contains articles on many of the points above.

Software and code

Policy information about availability of computer code

Data collection	Images for quantification purposes were acquired using the Leica Confocal microscope 63x/1.32 oil lens using LAS-AF Software (Leica). Analysis of the intensity of H3K9me3 or HP1 in the DAPI region was performed using an in-house written macro (ImageJ v2.1.0/1.53c).
Data analysis	Hi-C sequencing data was processed with Hi-C-Pro 2.9. We performed loop calling with HICCUPs 0.9. Hi-C data analysis was performed with GENOVA (github.com/deWitLab/GENOVA). Mapping of ChIPseq data was performed with bowtie 2.3.4.130 to hg19. We performed peak calling with MACS2 2.1.131. ChIPseq alignment plots were created with deeptools 3.0.0.<br>RNAseq data was mapped with TopHat 2.1.133 and count-tables were generated with HTseq34 with the stranded=reverse setting using the Gencode v27lift37 gene-build. TPMs were calculated with DESeq2 1.18.135

For manuscripts utilizing custom algorithms or software that are central to the research but not yet described in published literature, software must be made available to editors/reviewers. We strongly encourage code deposition in a community repository (e.g. GitHub). See the Nature Research guidelines for submitting code & software for further information.

Data

Policy information about availability of data

All manuscripts must include a data availability statement. This statement should provide the following information, where applicable:

- Accession codes, unique identifiers, or web links for publicly available datasets
- A list of figures that have associated raw data
- A description of any restrictions on data availability

Data has been deposited at GEO under accession GSE125672.

Field-specific reporting

Please select the one below that is the best fit for your research. If you are not sure, read the appropriate sections before making your selection.

- [x] Life sciences
- [ ] Behavioural & social sciences
- [ ] Ecological, evolutionary & environmental sciences

For a reference copy of the document with all sections, see nature.com/documents/nr-reporting-summary-flat.pdf

Life sciences study design

All studies must disclose on these points even when the disclosure is negative.

Sample size	For the Hi-C data two replicates were generated. Replicates were highly similar and combined into one dataset. For RNAseq experiments triplicate libraries were generated. No sample size calculations were performed. Sample sizes were chosen based on common standards of the field.
Data exclusions	No data was excluded.
Replication	RNAseq experiments were performed at in triplicate for any given cell line. All attempts were succesful.
Randomization	No randomization was performed.
Blinding	Blinding is not relevant to the current study because only machine measurements were used.

Reporting for specific materials, systems and methods

We require information from authors about some types of materials, experimental systems and methods used in many studies. Here, indicate whether each material, system or method listed is relevant to your study. If you are not sure if a list item applies to your research, read the appropriate section before selecting a response.

Materials & experimental systems

n/a	Involved in the study
[ ]	[x] Antibodies
[ ]	[x] Eukaryotic cell lines
[x]	[ ] Palaeontology
[x]	[ ] Animals and other organisms
[x]	[ ] Human research participants
[x]	[ ] Clinical data

Methods

n/a	Involved in the study
[ ]	[x] ChIP-seq
[x]	[ ] Flow cytometry
[x]	[ ] MRI-based neuroimaging

Antibodies

Antibodies used	The following antibodies were used for western blots: WAPL (A-7, sc-365189, Santa Cruz), HSP90 (F-8, sc-13119 Santa Cruz), MED12 (A300-774A, Bethyl), CCNC (ab85927, Abcam), CTCF (ab70303, Abcam), Actin (ab6276 Abcam) and Tubulin (T5168, Sigma-Aldrich). All primary antibodies were used at a 1:1000 dilution, except for Tubulin 1:4000.<br>For immunofluorescence: H3K9Me3 (ab8898, Abcam) or HP1alpha (Clone 15 19s2, Upstate/MilliporeSigma) antibody at a 1:1000 dilution.<br>For ChIP: SCC1 (ab992, Abcam), CTCF (3418S, Cell Signaling), H3K4me3 (PAB-003-050, Diagenode), H3K4me1 (PAB-037-050, Diagenode), H3K36me3 (MAB-183-050, Diagenode), H3K27me3 (PAB-195-050, Diagenode), H3K9me3 (PAB-193-050, Diagenode), MED12 (A300-774A, Bethyl)
Validation	Validation information can be found at the following websites for the following proteins:<br>WAPL: https://www.scbt.com/p/wapl-antibody-a-7<br>HSP90: https://www.scbt.com/p/hsp-90alpha-beta-antibody-f-8<br>MED12: https://www.fortislife.com/products/primary-antibodies/rabbit-anti-med12-antibody/BETHYL-A300-774<br>CCNC: https://www.abcam.com/cyclin-c-antibody-ab85927.html<br>CTCF: https://www.abcam.com/ctcf-antibody-ab70303.html<br>Actin: https://www.abcam.com/beta-actin-antibody-ac-15-ab6276.html<br>Tubulin: https://www.sigmaaldrich.com/NL/en/product/sigma/t5168<br>H3K9Me3: https://www.abcam.com/histone-h3-tri-methyl-k9-antibody-chip-grade-ab8898.html<br>HP1 alpha: https://www.fishersci.com/shop/products/anti-hp1-clone-15-19s2-millipore-upstate/50171770<br>SCC1: https://www.abcam.com/rad21-antibody-ab992.html<br>CTCF: https://www.cellsignal.com/products/primary-antibodies/ctcf-d31h2-xp-rabbit-mab/3418<br>H3K4Me3: https://www.diagenode.com/en/p/h3k4me3-polyclonal-antibody-premium-50-ug-50-ul

H3K4Me1: https://www.diagenode.com/en/p/h3k4me1-polyclonal-antibody-classic-50-ug-18-ul
H3K36Me3: https://www.diagenode.com/en/documents/datasheet-h3k36me3-mab-183-050
H3K27me3: https://www.diagenode.com/en/p/h3k27me3-polyclonal-antibody-premium-50-mg-27-ml
H3K9me3: https://www.diagenode.com/en/documents/datasheet-h3k9me3-pab-193-050

Eukaryotic cell lines

Policy information about cell lines

Cell line source(s)	Hap1 cells: Carette et al., Nature 2011 a gift from the authors.
Authentication	Karyotyping and western blot analyses. Knock-outs were confirmed by Western Blot.
Mycoplasma contamination	All cell lines were negative for mycoplasma contamination.
Commonly misidentified lines (See ICLAC register)	No commonly misidentified line was used.

ChIP-seq

Data deposition

☒ Confirm that both raw and final processed data have been deposited in a public database such as GEO.

☒ Confirm that you have deposited or provided access to graph files (e.g. BED files) for the called peaks.

Data access links
May remain private before publication.

Go to https://www.ncbi.nlm.nih.gov/geo/query/acc.cgi?acc=GSE125672
Enter token qlyhamqyvxohlyv into the box.

Files in database submission

GSM3579008_4557_1_Hap1_K4mono_CCGTCC_S1_peaks.narrowPeak.gz
GSM3579012_4590_11_4_K4mo_DKO_chipseq_TAGCTT_S84_L008_peaks.narrowPeak.gz
GSM3579014_4590_1_2_K4mo_Wapl_chipseq_CGATGT_S74_L006_peaks.narrowPeak.gz
GSM3579015_DKO_K9_minus100kbroad.bed.gz
GSM3579019_WAPL_K9_minus100kbroad.bed.gz
GSM3579022_4590_6_3_K4mo_Med12_chipseq_CAGATC_S79_L007_peaks.narrowPeak.gz
GSM3579024_MED12_K9_minus100kbroad.bed.gz
GSM3579026_WT_K9_minus100kbroad.bed.gz
CCNC_CTCF_peaks.narrowPeak
CCNC_SCC1_peaks.narrowPeak
CTCF_MED12_peaks.narrowPeak
CTCF_WT_peaks.narrowPeak
SCC1_MED12_peaks.narrowPeak
SCC1_WT_peaks.narrowPeak
WT_MED12_noBG.bed
GSM5570288_4590_10_3_K36tri_Med12_chipseq_ATCACG_S83_L007.bam_peaks.broadPeak.gz
GSM5570289_4590_7_3_K4tri_Med12_chipseq_CTTGTA_S80_L007_peaks.narrowPeak.gz
GSM5570290_4590_9_3_K27tri_Med12_chipseq_GTGAAA_S82_L007_peaks.narrowPeak.gz
GSM5570291_merged_K27tri_peaks.narrowPeak.gz
GSM5570286_4557_2_Hap1_K4tri_GTGAAA_S2_peaks.narrowPeak.gz
GSM5570287_4557_5_Hap1_K36tri_CGATGT_S5.bam_peaks.broadPeak.gz

Genome browser session (e.g. UCSC)

https://genome.ucsc.edu/s/robinhweide/CKM_haarhuis

Methodology

Replicates

No replicates were performed for the ChIPseq (n=1)

Sequencing depth

	reads	reads uniquely mapped
H3K4me1 WT	47968697	47167854
H3K4me1 MED12	37853636	37112517
H3K4me1 WAPL	38230393	37424292
H3K4me1 DKO	42861419	41996564
H3K9me3 WT	107939765	102169221
H3K9me3 MED12	90689208	85875210
H3K9me3 WAPL	89468188	83672296
H3K9me3 DKO	89247752	84069786
SCC1 WT	20882936	20441958

SCC1 MED12 21680259 21281073
SCC1 CCNC 20451607 20066494
MED12 WT 38953698 38141362
CTCF WT 22556481 22207251
CTCF MED12 18292327 17650638
CTCF CCNC 20577084 20096714
H3K4me3 WT 24474905 23841024
H3K4me3 MED12 30915587 30007460
H3K27me3 WT 91963375 87959134
H3K27me3 MED12 71125320 68617412
H3K36me3 WT 40155268 39664965
H3K36me3 MED12 46760825 45972158

Antibodies

SCC1 (ab992, Abcam), CTCF (3418S, Cell Signaling), MED12 (A300-774A, Bethyl), antibodies from Diagenode: PAB-003-050 for H3K4me3, PAB-037-050 for H3K4me1, MAB—183-050 for H3K36me3, PAB-195-050 for H3K27me3 and PAB-193-050 for H3K9me3.

Peak calling parameters

We performed peak calling with MACS2 2.1.131 for SCC1, CTCF and H3K4me1 with standard settings. For H3K9me3 we performed peak calling in advanced mode using the following settings -l 500 -g 65 --cutoff-analysis 2.25.

Data quality

N peaks > 5FC N peaks > -log10(0.05)
H3K4me1 WT 12862 166392
H3K4me1 MED12 5358 115991
H3K4me1 WAPL 25105 156397
H3K4me1 DKO 6855 131806
SCC1 WT 34304 43128
SCC1 MED12 26065 32571
SCC1 CCNC 28885 36637
CTCF WT 57812 67132
CTCF MED12 49737 56129
CTCF CCNC 54577 65989
MED12 WT 15022 33108
H3K9me3 WT 27403 NA
H3K9me3 MED12 4976 NA
H3K9me3 WAPL 58337 NA
H3K9me3 DKO 41370 NA
H3K4me3 WT 18279 26647
H3K4me3 MED12 19970 29517
H3K27me3 WT 2427 7124
H3K27me3 MED12 2224 4081
H3K36me3 WT 44 16503
H3K36me3 MED12 50 14039

Software

We performed peak calling with MACS2 2.1.131 for SCC1, CTCF and H3K4me1 with standard settings. For H3K9me3 we performed peak calling in advanced mode using the following settings -l 500 -g 65 --cutoff-analysis 2.25.
