# Supplementary figures and images for: A Mediator-cohesin axis controls heterochromatin domain formation
Source: Nat Commun. 2022 Feb 8;13:754. doi: 10.1038/s41467-022-28377-7 (PMC8826356; doi:10.1038/s41467-022-28377-7)

Figure 2b

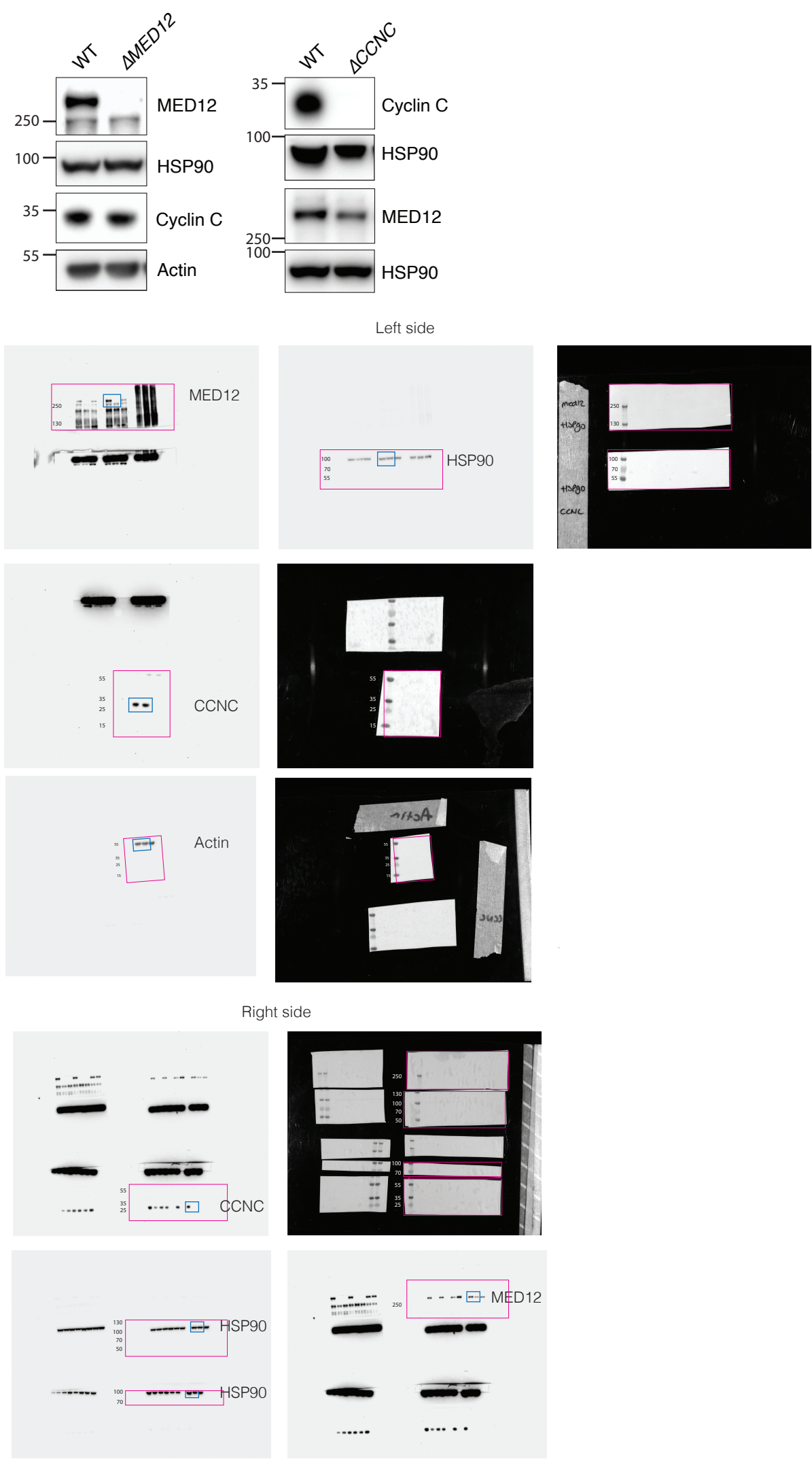

Supplement: Supplementary file 3 — Source Data [file 41467_2022_28377_MOESM3_ESM.zip › source_data/Figure2b.pdf]

Figure 5a

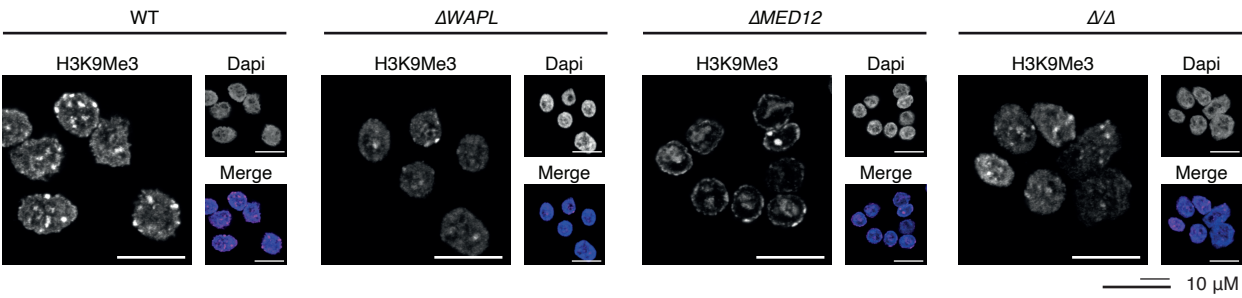

Raw images

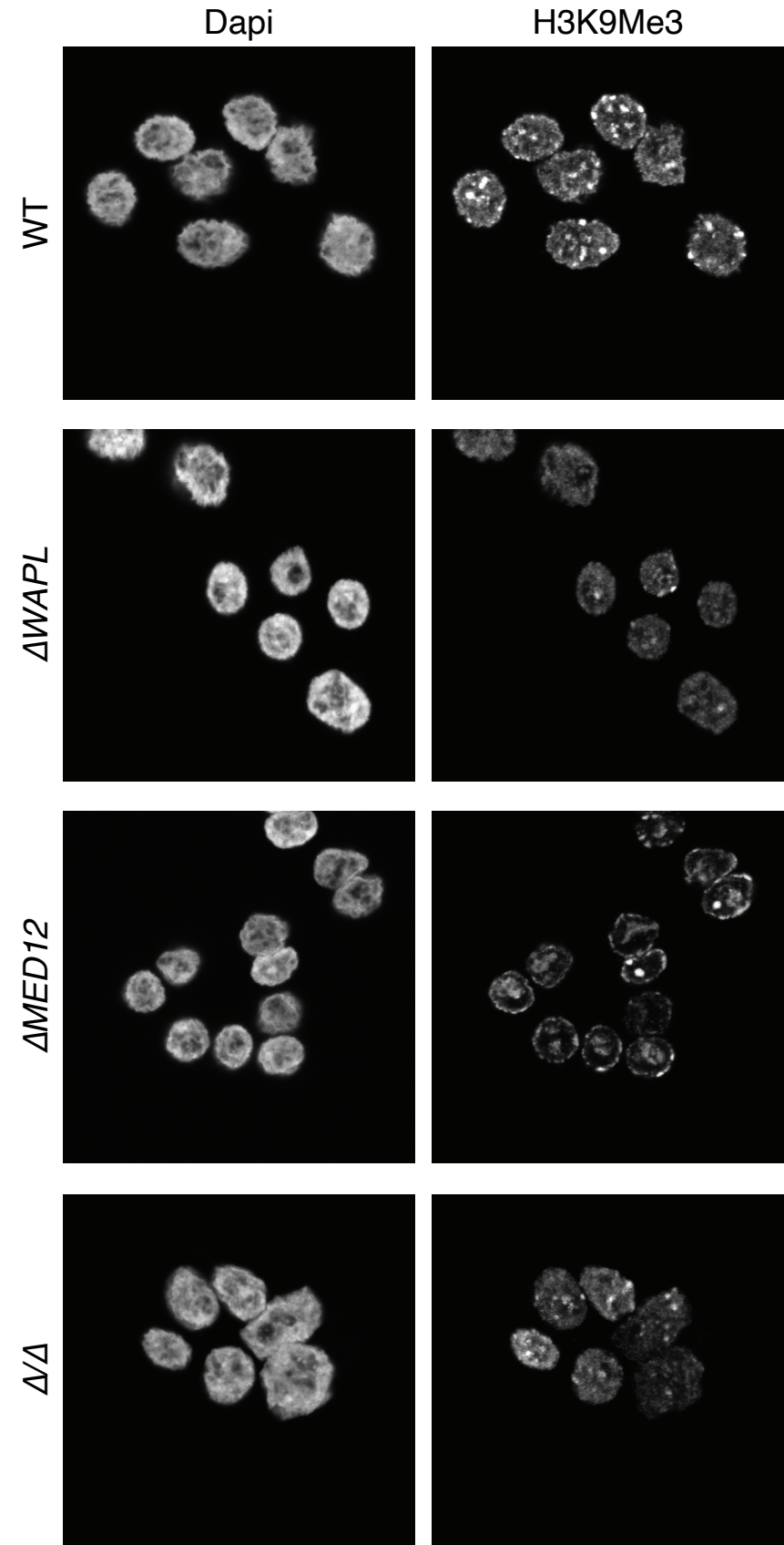

Supplement: Supplementary file 3 — Source Data [file 41467_2022_28377_MOESM3_ESM.zip › source_data/Figure5a.pdf]

Figure ED 4d

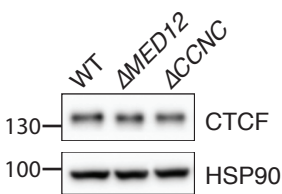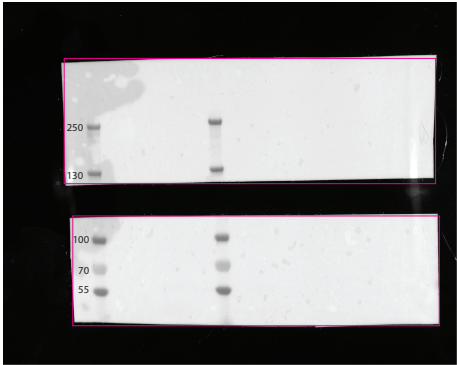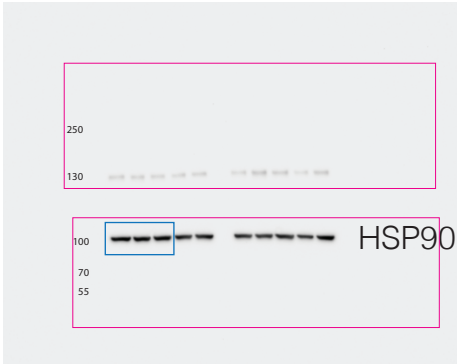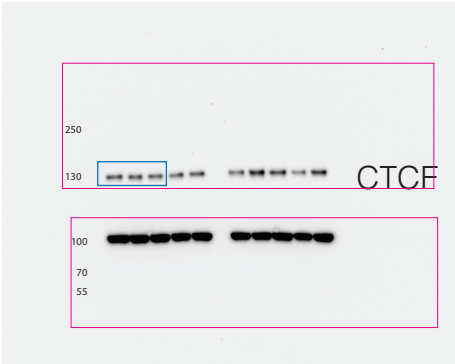

Supplement: Supplementary file 3 — Source Data [file 41467_2022_28377_MOESM3_ESM.zip › source_data/SuppFigure4d.pdf]

Figure ED 5a

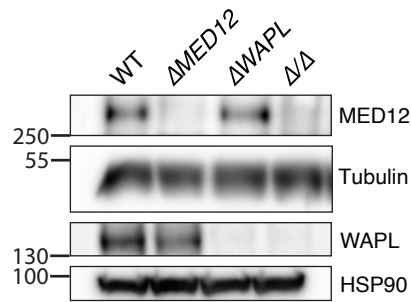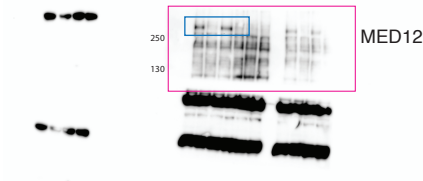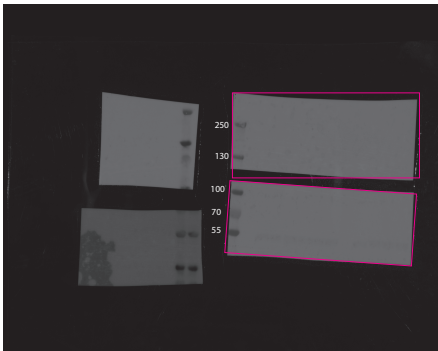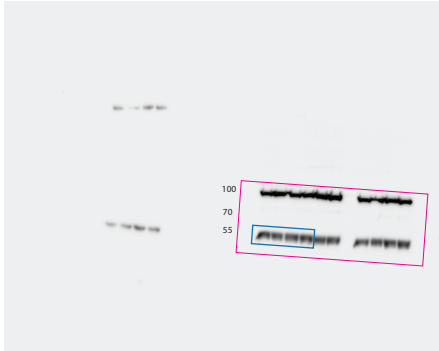

Tubulin

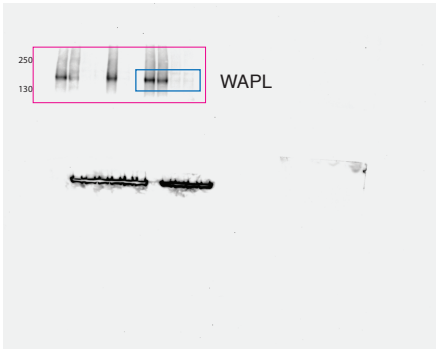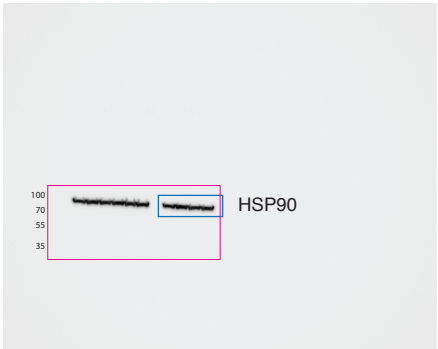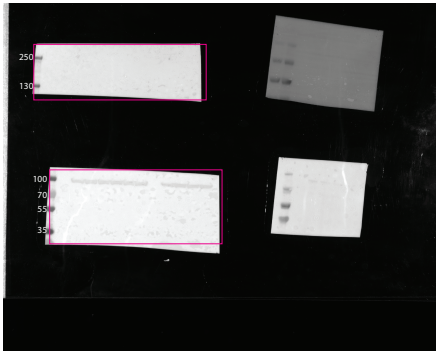

Supplement: Supplementary file 3 — Source Data [file 41467_2022_28377_MOESM3_ESM.zip › source_data/SuppFigure5a.pdf]

Figure 2h

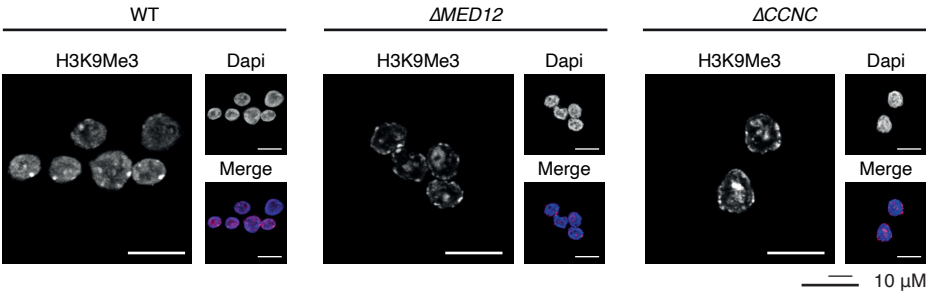

Raw images

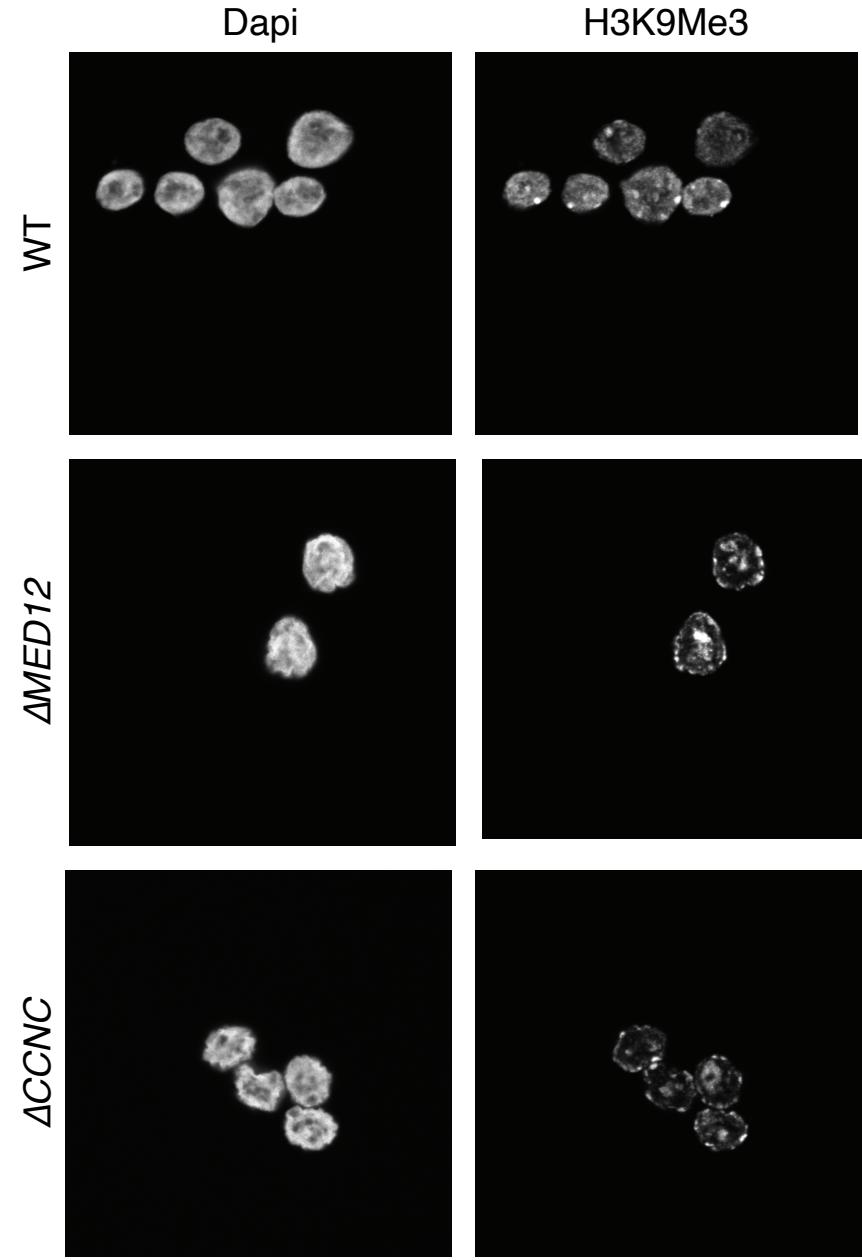

Supplement: Supplementary file 3 — Source Data [file 41467_2022_28377_MOESM3_ESM.zip › source_data/Figure2h.pdf]

Figure ED 2f

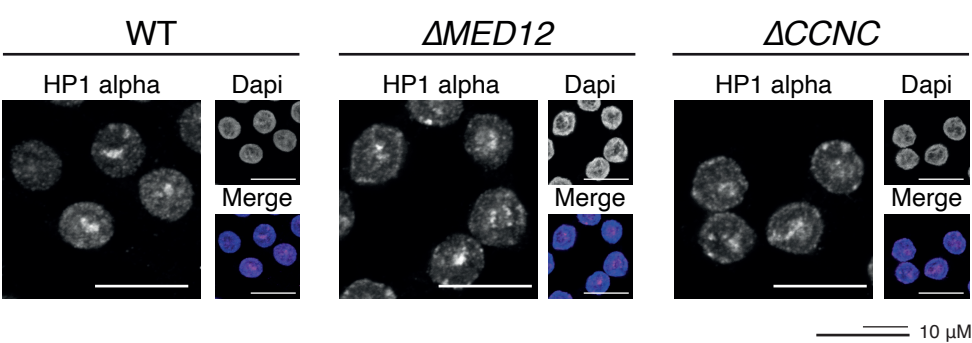

Raw images

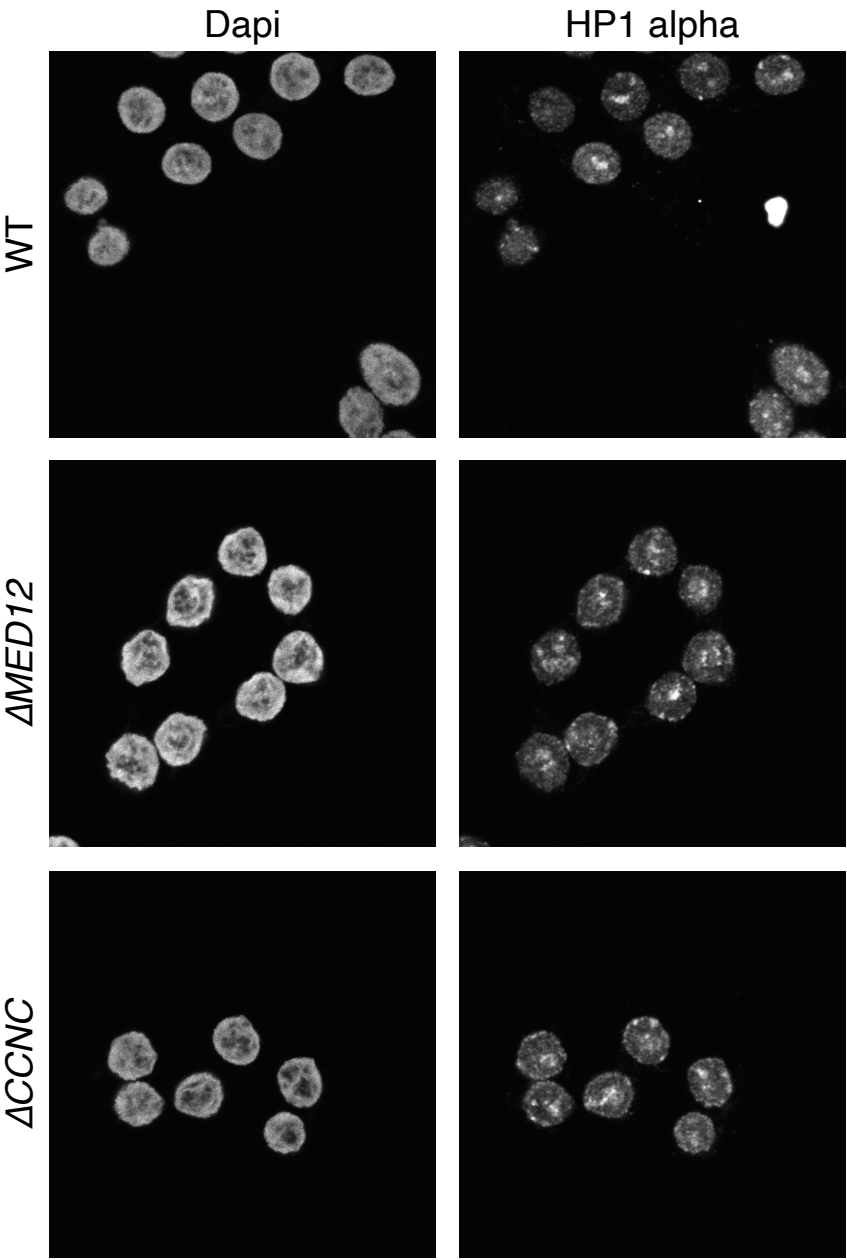

Supplement: Supplementary file 3 — Source Data [file 41467_2022_28377_MOESM3_ESM.zip › source_data/SuppFigure2f.pdf]
